# Fatigue Cracking Resistance of Crumb Rubber-Modified Asphalt: Mechanisms, Testing Methods, and Multiscale Performance Evaluation from Binder to Field

**DOI:** 10.3390/polym18192451

**Published:** 2026-10-08

**Authors:** Anil Rai, Sushmit Sharma Bhattarai, Hyunhwan Kim, Moon-Sup Lee, Soon-Jae Lee

**Affiliations:** 1 Department of Engineering Technology, Texas State University, San Marcos, TX 78666, USA; vst33@txstate.edu (A.R.); k_h82@txstate.edu (H.K.); sl31@txstate.edu (S.-J.L.); 2 Construction Management, Department of Construction Management, U.A. Whitaker College of Engineering, Florida Gulf Coast University, Fort Myers, FL 33965, USA; ssharmabhattarai@fgcu.edu; 3 Korea Institute of Civil Engineering and Building Technology, Goyang-si 10223, Gyeonggi-do, Republic of Korea

**Keywords:** crumb rubber-modified asphalt, fatigue cracking, linear amplitude sweep, viscoelastic continuum damage, field performance, mechanistic modeling

## Abstract

Fatigue cracking is a load-related distress limiting flexible pavement service life. This review evaluates 121 studies on the fatigue resistance of crumb rubber-modified asphalt (CRMA) across binder, mixture, modeling, and field scales. CRMA improves fatigue resistance through rubber–asphalt interaction and the elastic contribution of undissolved particles; adequate swelling, dispersion, and bonding slow crack propagation, whereas weak interaction or aging-related interface weakening promotes localized damage. Optimum rubber contents and particle sizes vary with damage stage, material scale, dosage basis, and processing. The benefit is strain-dependent, greater at 2.5% than 5.0%, and rankings may reverse between amplitudes. Linear viscoelastic parameters poorly characterize progressive damage in highly modified binders; linear amplitude sweep testing with viscoelastic continuum damage analysis is more informative but sensitive to the failure criterion. The benefit narrows with evaluation scale, though not uniformly: binder-level improvements reach 116 times the control at 2.5% strain and 45 times at 5.0%, mixture-scale improvements about 1.4 to 10 times (upper bound provisional), and field performance comparable to or moderately better than modified controls. CRMA is a viable alternative to polymer-modified asphalt under specific conditions; equivalence rather than superiority is defensible. Standardized failure criteria, phase-resolved aging procedures, protocols representing healing, and validated multi-scale relationships are needed.

## 1. Introduction

Fatigue cracking is one of the major load-related distresses limiting the service life of flexible asphalt pavements. Under repeated traffic loading at intermediate service temperatures, cyclic stresses and strains progressively damage the binder-rich phase, leading to microcrack initiation, growth, and propagation. The binder controls the early stages of fatigue damage, making its response under repeated loading critical for understanding crack development and improving asphalt pavement durability [1,2]. At the same time, the disposal of end-of-life tires remains a serious environmental challenge. Incorporating tire-derived crumb rubber into asphalt provides an established recycling approach that reduces tire waste and can improve elasticity, deformation recovery, and resistance to rutting and cracking [3,4,5,6]. These environmental and performance benefits have encouraged the use of crumb rubber-modified asphalt through wet, dry, terminal-blend, and related incorporation processes [7,8].

The performance of crumb rubber-modified asphalt has been attributed primarily to the physical and physicochemical interactions between crumb rubber and the asphalt binder. During wet processing, rubber particles absorb lighter asphalt fractions, swell, and form a rubber-rich elastic structure within the binder, while heat and shear may promote partial desulfurization and depolymerization that release rubber-derived constituents into the asphalt phase [9,10]. These changes can increase elastic recovery, improve energy dissipation and slow the accumulation of fatigue damage. The modification mechanism is generally explained by two complementary effects. The particle effect arises from the elastic and filler-like contribution of undissolved rubber particles, whereas the interaction effect results from swelling and material exchange at the rubber–asphalt interface [11].

Although crumb rubber generally improves fatigue resistance, the extent of improvement varies considerably among studies. Increasing rubber content can promote a denser elastic network and greater resistance to damage, but excessive dosages may increase viscosity, reduce storage stability, and complicate mixing and compaction [7]. Particle size also affects fatigue performance, since larger particles may retain a stronger particle-level elastic response, whereas finer particles provide greater surface area and interact more rapidly with the asphalt phase [9,11]. The incorporation method is another important factor because wet, dry, and terminal-blend processes differ in the extent of rubber–asphalt interaction, affecting workability, storage stability, and fatigue resistance [6,8]. Fatigue performance may also vary with strain level. For instance, terminal-blend modification produced clearer improvements at lower strain, with limited benefit under higher-strain conditions [12]. The method used to evaluate fatigue performance can influence the reported results.

The conventional Superpave parameter, |G*|sinδ, is obtained under linear viscoelastic conditions and does not directly reflect the evolution of damage during repeated loading [1,2,13,14]. Damage-based methods, including the linear amplitude sweep (LAS) test and viscoelastic continuum damage (VECD) analysis, provide a more detailed characterization of damage evolution and strain-dependent fatigue life [2,13]. However, their outcomes remain influenced by testing conditions and analytical procedures, and their ability to represent mixture- and field-scale cracking has not yet been fully established [2,13,14]. The fatigue behavior of CRMA is also affected by aging through asphalt oxidation, rubber degradation, and changes at the rubber–asphalt interface. These processes generally increase stiffness and reduce fatigue life, although the extent of deterioration varies with material composition and processing conditions [14]; reported fatigue outcomes for aged CRMA nevertheless remain inconsistent, a divergence attributed largely to the choice of evaluation indicator [15]. In addition, laboratory testing and aging protocols may not fully reproduce the combined environmental and loading conditions experienced during pavement service [15], limiting the direct translation of binder-level findings to mixture- and pavement-scale performance [1,2].

Although previous reviews have addressed crumb rubber production, interaction mechanisms, rheological behavior, aging, sustainability, and overall pavement performance [3,4,6,7,9,15], fatigue-specific evidence remains distributed across studies examining different materials, testing methods, aging conditions, processing techniques, field performance, and modeling approaches. Consequently, an integrated understanding of how crumb rubber modification and fatigue evaluation methods relate to binder-, mixture-, and pavement-scale performance remains limited. This limitation makes direct comparison among studies difficult and restricts the identification of the material characteristics, evaluation methods, and performance indicators most appropriate under different testing and performance conditions for assessing CRMA fatigue resistance.

To address this gap, the objective of this review is to synthesize and critically evaluate existing evidence on the fatigue cracking resistance of CRMA. The review discusses fatigue cracking mechanisms and the elastic recovery behavior of CRMA and assesses conventional dynamic shear rheometer (DSR) fatigue parameters, LAS testing, and VECD analysis. It further evaluates the effects of aging and crumb rubber particle size on fatigue performance and compares wet- and dry-process modification. Mixture-level testing, field observations, and mechanistic modeling are also examined to assess whether binder-scale findings are consistent with mixture- and pavement-scale performance. Earlier reviews have generally examined CRMA performance within a single evaluation scale. The present review instead follows the reported magnitude of the fatigue benefit across the binder, mixture, mechanistic, and field scales, and it examines how far the values reported at one scale carry over to the next. The review is therefore organized around a single distress examined across several evaluation scales, rather than around an individual material variable, such as crumb rubber particle size. Particle size is treated here only in so far as it bears on fatigue behavior. A synthesis organized around that variable would address a different and complementary question. Finally, the review identifies methodological limitations and areas requiring further investigation to improve the reliability and field relevance of CRMA fatigue assessment.

## 2. Methodology

A structured critical review was conducted to identify and critically evaluate studies on the fatigue cracking resistance of CRMA. The review adopts the identification, screening, and selection logic used in PRISMA for transparency of study selection, but it is a critical rather than a fully systematic review: the evidence was synthesized qualitatively, and neither meta-analysis nor formal risk-of-bias scoring was undertaken. This distinction is stated explicitly because the heterogeneity of materials, test protocols, and failure criteria across the reviewed studies precludes pooled quantitative estimation.

Scopus was used as the primary database, while Google Scholar, ScienceDirect, and the ASCE Library were used as supplementary sources. Publications issued between 1990 and the final search date were retrieved using combinations of terms related to CRMA, fatigue cracking, DSR, LAS, VECD, aging, crumb rubber content and particle size, wet and dry processes, field performance, and mechanistic modeling. The final search of that stage was performed in May 2026. During review, the search strategy was found not to reach the mixture and field scales reliably, and it was replaced by three scale-specific strings used as a union. The revised strings, the database and date against which each was executed, the number of records returned, and a recall test of both the original and the revised strategy are reported in Appendix A.

The following elements of the search and screening procedure are reported to improve the transparency and reproducibility of the study-selection process. The Boolean search strategy used to guide the literature search is provided in Appendix A, with the search syntax adapted as necessary to meet the requirements of each database. Only studies published in English were considered for inclusion in the review. Theses, dissertations, and conference proceedings were excluded from the primary evidence synthesis; however, selected authoritative agency reports and standard test methods were retained as supplementary sources when they provided relevant field evidence or methodological guidance. Title and abstract screening, followed by full-text eligibility assessment, was conducted using predefined inclusion and exclusion criteria. No formal study-quality or risk-of-bias scoring instrument was applied; a qualitative grading of the reviewed studies by completeness of experimental design and level of variable control is reported in Appendix B. Differences in study design, testing methods, evaluation criteria, and relevance to fatigue performance were considered qualitatively when interpreting and comparing the findings. For each included study, where applicable, the following information was extracted: crumb rubber characteristics, processing and aging conditions, test method, evaluation scale, loading conditions, comparator material, and reported fatigue or cracking performance. For field studies, traffic or exposure conditions and observed pavement performance were considered, while modeling studies were characterized by the modeling approach, calibration data, and predicted fatigue performance. The extracted evidence was then classified according to the three evidence tiers described below.

**Figure 1:**
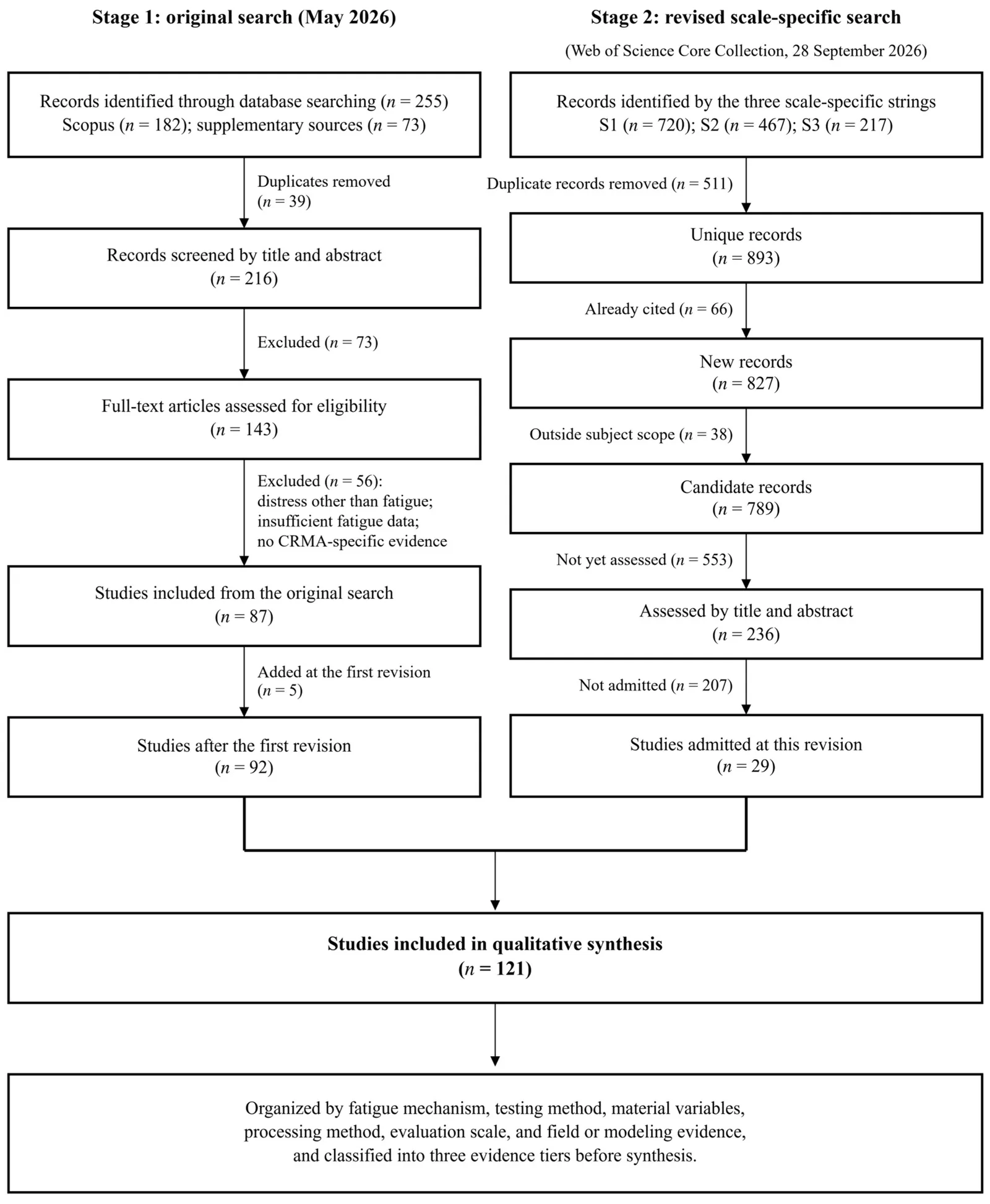
Flow diagram of the literature identification, screening, and selection process. Stage 1 is the search reported in the original submission. Stage 2 is the revised scale-specific search executed during revision, in which S1, S2, and S3 denote the binder-, mixture-, and field-scale strings defined in Appendix A. Screening of the Stage 2 candidate records is not complete, and the records not yet assessed are shown as such. CRMA, crumb rubber-modified asphalt.

The search identified 255 records, including 182 from Scopus and 73 from supplementary sources. After removing 39 duplicates, 216 publications were screened by title and abstract, and 143 were assessed in full text. Studies were included when they directly addressed fatigue mechanisms, binder- or mixture-level fatigue evaluation, aging, crumb rubber characteristics, processing methods, field cracking performance, or fatigue-related modeling. Studies focusing on rutting, moisture damage, low-temperature cracking, or other pavement distresses were excluded unless they provided evidence directly relevant to fatigue behavior. Monotonic cracking studies were considered only as supplementary evidence of crack initiation or propagation and were interpreted separately from repeated-load fatigue results. Following full-text evaluation, 87 studies were selected for qualitative synthesis and comparative evaluation. Five further studies at the mixture and field scales were identified during the first revision through supplementary scale-specific searches and were added to the synthesis, giving 92 studies. At the present revision, the revised strategy was executed formally, returning 893 unique records, of which 66 were already cited; a total of 789 of the remainder were retained as candidates after applying the exclusion criteria above, 236 were assessed at the title and abstract levels, and 29 studies were admitted, giving us 121 studies for the synthesis. Screening of the remaining candidate records is not complete, and the limitations of the search and of the screening carried out so far are stated in Appendix A. A small number of further sources are cited outside the synthesis. These are foundational references on viscoelastic continuum damage theory and standard test methods, together with two sources on emerging molecular- and nanoscale characterization methods cited in the research outlook. They support the description of the testing and analysis procedures. None of them were retrieved through the search process, and none were counted among the studies selected through screening. The selection process that produced the 87 screened studies of the original search is summarized in Figure 1; the Figure depicts that stage only, and the yields of the revised search are reported in Appendix A.

Because the reviewed studies employed different classes of tests, the evidence was classified into three tiers before synthesis, and this distinction is retained throughout the paper. Tier 1, direct repeated-load fatigue evidence, comprises LAS, time sweep, direct-tension simplified VECD (S-VECD), four-point bending, and cyclic indirect tensile fatigue testing. Tier 2, supporting cracking and fracture evidence, comprises monotonic fracture tests, reflective-cracking assessments, and fracture-energy indices; these characterize cracking tolerance but do not reproduce the progressive damage accumulation of repeated loading. Tier 3, indirect rheological indicators, comprises the Superpave fatigue parameter, the Glover–Rowe parameter, elastic recovery, and parameters derived from multiple stress creep and recovery tests; these describe the undamaged or lightly damaged response and are treated here as screening information only. Quantitative fatigue-life ranges reported in this review are intended to rest on Tier 1 evidence; where a value derives from Tier 2 or Tier 3 evidence, this is stated at the point of use.

The selected studies were organized according to fatigue mechanism, testing method, material variables, processing method, evaluation scale, and field or modeling evidence. The reviewed studies showed considerable variation in material composition, testing conditions, fatigue evaluation criteria, and reported performance measures. Therefore, the findings were synthesized qualitatively to identify consistent trends, interpret differences among reported findings, and assess relationships across binder, mixture, mechanistic, and field scales.

## 3. Fatigue Cracking Mechanism

Fatigue cracking is one of the primary distress modes in flexible asphalt pavements, and understanding its mechanism at the binder level is fundamental to evaluating the performance of crumb rubber-modified asphalt binders. Repeated traffic loading subjects asphalt to cyclic shear and tensile deformation, causing localized stress to accumulate within the viscoelastic matrix and gradually reducing the material’s ability to recover, which leads to internal damage and microcrack formation [1,2]. Therefore, fatigue resistance is not governed by stiffness alone, but also by the capacity to relax stress, dissipate energy, and maintain structural integrity under prolonged cyclic loading [16]. As damage progresses, these microcracks accumulate and merge, leading to the crack propagation phase, during which a crack front advances steadily through the matrix. Fatigue crack density evolution and modified Paris’ law analysis have shown that this process develops through distinct crack-initiation and steady crack-growth stages under controlled-strain loading [17]. These stages highlight the importance of binder modification strategies that can delay crack initiation and reduce the rate of crack propagation [2].

The mechanism described above is that of repeated-load fatigue damage. Temperature, environmental cycling, and loading rate do not constitute that mechanism; they modify the susceptibility of the binder to it, and are distinguished from it here. Low-temperature thermal cracking and freeze–thaw damage are separate distress modes and are not treated as fatigue cracking in this review. At low temperatures, asphalt becomes stiffer and loses its ability to relax stress, which concentrates stress at existing crack tips and accelerates microcrack growth once cyclic loading is applied [18,19]. Freeze–thaw cycling introduces repeated thermal stresses that weaken the matrix and promote crack initiation under subsequent traffic loading [20]. Loading rate acts on the fatigue mechanism itself: at lower loading frequencies, each load cycle lasts longer, allowing more time for crack growth and damage accumulation. Fatigue life has been reported to decrease with increasing temperature and decreasing loading frequency [21]. Trends of this kind are sensitive to the loading-control mode and should be read together with the test conditions under which they were obtained, because stress-controlled and strain-controlled testing can yield opposite temperature trends for the same material. Reported temperature and frequency effects are therefore not transferable between control modes unless the mode is stated. Furthermore, advanced binder-level testing using time sweep, linear amplitude sweep, and VECD-based methods has established that fatigue cracking involves nonlinear stiffness degradation that cannot be adequately captured by the traditional |G*|sinδ parameter alone [1,2].

### 3.1. Fatigue Cracking Resistance Mechanisms of CRMA Binders

The addition of crumb rubber modifies the physical and chemical structure of the asphalt binder through a swelling mechanism, in which rubber particles absorb light aromatic components from the base bitumen, expand in volume, and form a gel-like elastic network within the binder matrix [9,10]. The swelling of crumb rubber within the asphalt matrix can stiffen the surrounding binder and restrict particle mobility, thereby reducing the possibility of microcrack formation under repeated loading [5]. Beyond this local stiffening effect, crumb rubber can also shift the material response from relatively brittle fracture toward a more ductile damage process. Rubber-modified mastics exhibited greater post-peak deformation and stronger resistance to crack growth than unmodified and polymer-modified mastics, suggesting that dispersed rubber particles interrupt the crack path and increase the energy required for further propagation [19]. Singh et al. [11] further explained that the fatigue improvement of CRMA binders arises from both the particle effect, associated with the physical filler role of rubber particles, and the interaction effect, associated with swelling and physicochemical interaction at the rubber–asphalt interface. However, the contribution of each mechanism depends on rubber content and the degree of rubber–asphalt interaction. Under both controlled-strain and controlled-stress loading, increasing rubber content up to 40% progressively improved fatigue cracking resistance, indicating that a more developed rubber network can delay damage accumulation when adequate dispersion and interaction are achieved [17].

### 3.2. Elastic Recovery Effects of CRMA Binders

Elastic recovery is one of the most important mechanical improvements associated with crumb rubber modification and is closely related to fatigue cracking resistance. In asphalt binders, elastic recovery refers to the ability of the material to regain its original shape after the applied load is removed [22]. A higher recovery response indicates that a greater portion of the imposed strain can be recovered rather than retained as permanent viscous deformation, thereby reducing damage accumulation during repeated loading [23]. CRMA binders contribute to this response through the cross-linked polymer network retained within crumb rubber particles after tire vulcanization. This network promotes recoverable deformation as the rubber swells and partially degrades within the asphalt matrix [10]. When properly swollen and uniformly dispersed within the asphalt matrix, crumb rubber particles increase the recoverable component of cyclic deformation and reduce the accumulation of irreversible fatigue damage. The magnitude of this improvement can be substantial, as crumb rubber modification was reported to increase the elastic recovery of the base bitumen by approximately 339.9% [10].

This enhanced recovery response is also reflected in the rheological behavior of CRMA binders. Compared with unmodified base binders, CRMA binders generally exhibit higher percent recovery and lower non-recoverable creep compliance, indicating a greater ability to recover deformation after loading and reduce permanent strain accumulation. Although these parameters do not directly measure fatigue life, they provide supporting evidence of a binder’s ability to withstand repeated deformation with less residual damage. For instance, reacted and activated rubber (RAR)-modified binders exhibited improved elastic response, indicating enhanced ability to recover after deformation [24]. Similar behavior was observed in field-blended CRMA binders, which exhibited lower permanent deformation and higher elastic recovery than the corresponding base binder, supporting the practical relevance of the recovery benefits identified in laboratory studies [25]. In hybrid styrene–butadiene–styrene/crumb rubber (SBS/CR) binder systems, sulfur cross-linking strengthened the three-dimensional polymer network, and the SBS/CR/sulfur formulation provided the most balanced performance in terms of elastic recovery and resistance to fatigue damage [23].

Beyond immediate elastic recovery, CRMA binders may also exhibit healing-related recovery during rest periods between loading cycles. During unloading, the elastic restoring force of swollen rubber particles can help reduce crack opening and support partial recovery of the damaged binder structure. This healing response has been associated with rubber re-swelling, which contributes to the recovery of the initial complex modulus and fatigue performance after damage [26]. LAS-based healing evaluation has shown that recovery periods can extend fatigue life through structural reorganization and partial recovery of the damaged binder structure. This finding indicates that the fatigue benefit of CRMA binders is not limited to immediate elastic recovery but also includes time-dependent healing mechanisms that slow damage accumulation under repeated loading [27].

The extent of both immediate elastic recovery and healing-related recovery is strongly influenced by rubber content. Increasing the CR dosage generally promotes the development of a denser elastic network and improves fatigue life, although excessive rubber content can substantially increase viscosity and reduce storage stability [7,9,24,27]. However, this improvement does not increase indefinitely with rubber content. Although higher dosages can enhance resistance to crack initiation, CR contents above 4% by binder mass may reduce crack-propagation resistance when undissolved rubber particles act as local stress concentrators [28]. To address these limitations, researchers have explored composite modification systems. Adding terminal-blend rubber to an SBS-modified binder increased fatigue life by approximately four times compared with SBS modification [29]. Furthermore, CR/SBS composite binders matched or exceeded the fatigue resistance of the reference SBS-modified binder and also exhibited lower sensitivity to long-term aging, as reflected by reduced values of the Glover–Rowe parameter [30].

## 4. DSR-Based Fatigue Evaluation of CRMA Binders

At the binder scale, fatigue performance is evaluated by examining the response of CRMA under repeated loading. Since fatigue damage initiates and accumulates within the binder-rich phase before propagating through the asphalt mixture, reliable binder-level characterization is essential for evaluating and ranking modified binders, particularly CRMA systems. The dynamic shear rheometer (DSR) is the primary tool for binder fatigue evaluation because it captures both linear viscoelastic behavior and cyclic damage response under controlled testing conditions. DSR-based fatigue evaluation of CRMA binders can be broadly grouped into conventional rheological parameters, LAS testing, and VECD-based analysis.

### 4.1. DSR Fatigue Parameters

The Strategic Highway Research Program (SHRP) introduced the parameter |G*|sinδ as the standard Superpave fatigue factor to evaluate the cracking susceptibility of asphalt binders at intermediate service temperatures. Within the Superpave Performance Grading (PG) system, this parameter is evaluated on long-term aged binders, with a specification limit requiring |G*|sinδ ≤ 5000 kPa to ensure adequate fatigue resistance [2,13,16,31]. The parameter is obtained from a single-point DSR measurement under cyclic loading at small strain amplitude (typically 1%) and 10 rad/s, and is based on linear viscoelastic (LVE) response without consideration of damage accumulation [1,2,13]. For unmodified bitumen, lower |G*|sinδ is associated with improved fatigue resistance because both the modulus and the relative magnitude of the viscous component decrease together with energy dissipation per cycle. When crumb rubber is incorporated, a similar trend is initially observed. Oil-treated crumb rubber reduced |G*|sinδ by approximately 35% compared with neat bitumen [32]. Similarly, both 30–40 mesh and 60–80 mesh crumb rubber decreased |G*|sinδ through the combined effects of rubber–asphalt interaction and the filler-like behavior of rubber particles, with the particle effect being more dominant [11].

Despite its simplicity, the applicability of |G*|sinδ to CRMA and polymer-modified binders remains limited. The Superpave fatigue parameter relies on the LVE response and does not include damage evolution, so it cannot adequately capture the ability of CRMA binders to dissipate energy through ductile flow and crack pinning by dispersed rubber particles while maintaining structural integrity [1,2]. The Glover–Rowe (G-R) parameter, derived from frequency-sweep data at 15 °C and 0.005 rad/s, was proposed as a more sensitive alternative [33,34]. However, both |G*|sinδ and the G-R parameter were found to be insufficient for accurately characterizing the fatigue performance of long-term aged CRMA containing warm-mix additives [2]. A weak correlation was observed between |G*|sinδ and the measured fatigue life of degraded CRMA. Therefore, the LAS test was recommended as the preferred method for fatigue evaluation [14]. Similarly, LAS-based fatigue life provided a more meaningful fatigue ranking than |G*|sinδ for CRMA binders [13]. These findings indicate that |G*|sinδ and G-R are useful screening parameters, but damage-based methods such as LAS and VECD are required for reliable fatigue evaluation of CRMA binders.

### 4.2. LAS Test for CRMA Binders

**Figure 2:**
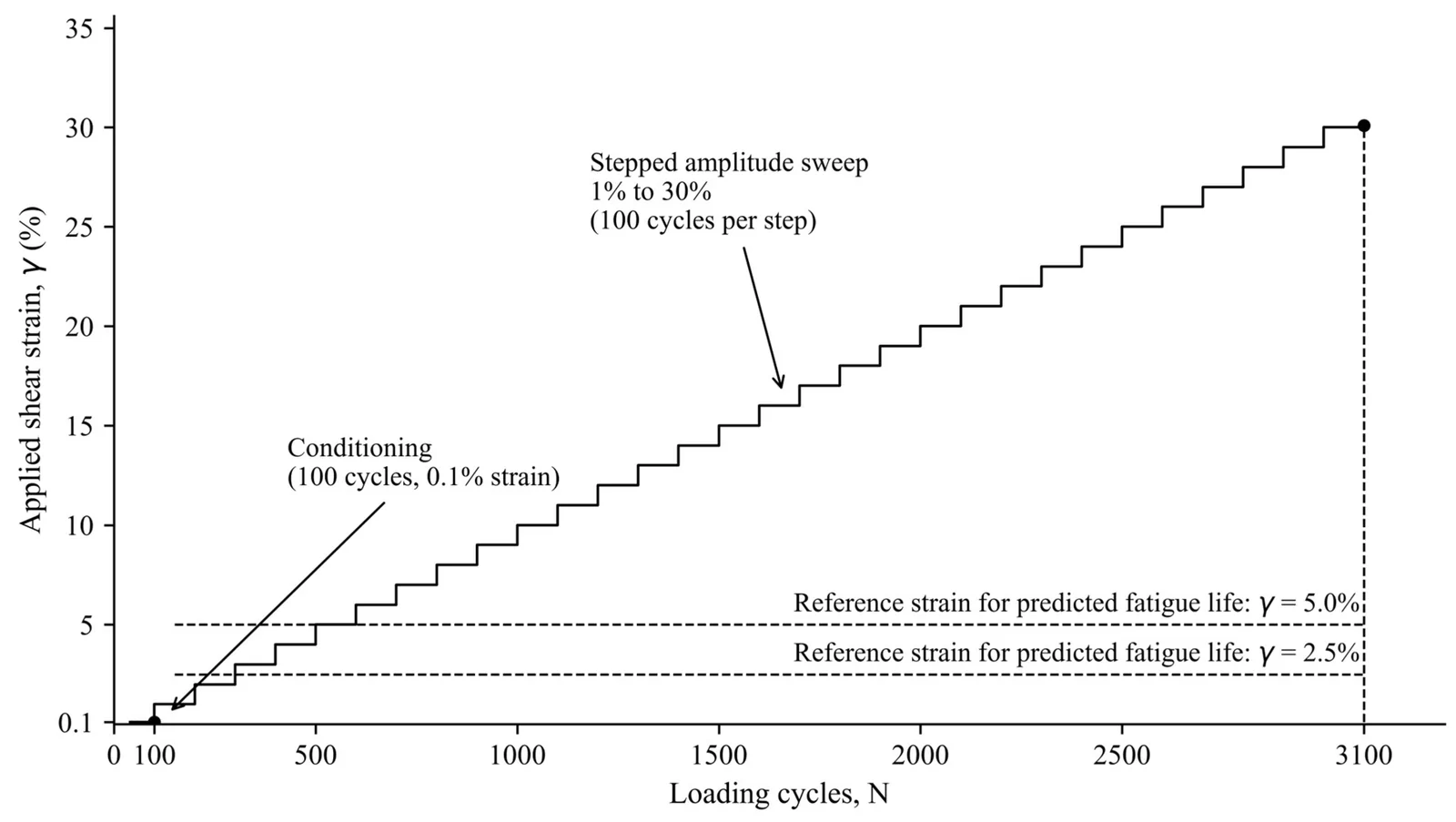
Schematic stepped strain profile of the LAS test [36].

The LAS test was proposed to address the limitation of the Superpave fatigue parameter |G*|sinδ, which is based only on linear viscoelastic response and does not directly characterize fatigue damage accumulation [35,36]. The LAS procedure has undergone several revisions across AASHTO standards. AASHTO TP 101-12 employed a stepped strain-loading protocol and defined failure as a 35% reduction in the undamaged |G*|sinδ [37]. TP 101-14 subsequently introduced a continuous linear strain ramp and adopted peak shear stress as the failure criterion [38]. AASHTO T 391-20 later readopted the stepped loading protocol and the 35% reduction criterion [36]. The test is performed at the intermediate PG temperature on PAV-aged binder and consists of two stages. A frequency sweep is first conducted at 0.1% strain, within the linear viscoelastic range, to characterize the undamaged response and obtain the parameter α used in the subsequent VECD-based analysis. An amplitude sweep is then applied at 10 Hz, beginning with conditioning at 0.1% strain followed by strain levels from 1% to 30% in 1% increments, with 100 cycles applied at each level. Fatigue life (N_f_) is reported at two reference strain levels (typically 2.5% and 5.0%) that are used as comparative lower- and higher-strain conditions and are commonly associated with thicker and thinner pavement structures, respectively [12,39]. The LAS strain profile is shown schematically in Figure 2.

In LAS/VECD analysis, the fatigue life of asphalt binders is commonly expressed as a power-law function of the maximum applied shear strain as:

Predicted fatigue life (N_f_) = A * (γ_max_)^−B^ (1)

where γ_max_ is the maximum expected binder strain and A and B are fitting parameters derived from VECD-based analysis of the LAS data [35,37]. Reporting at two strain levels is important for CRMA binders because the relative fatigue benefit of crumb rubber is highly strain-dependent.

**Figure 3:**
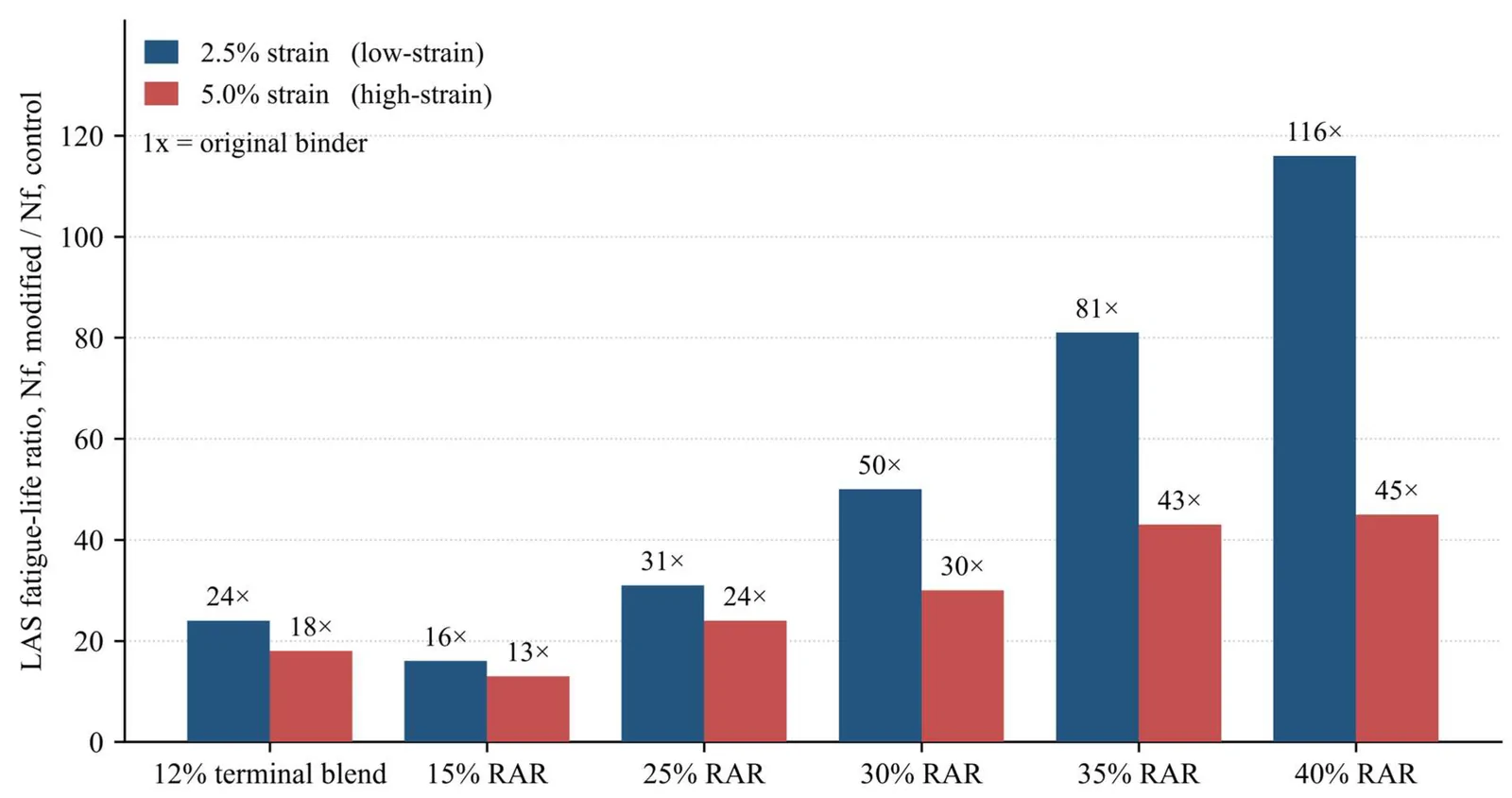
Reported LAS fatigue-life ratios of selected CRMA binder systems relative to their corresponding control binders at 2.5% and 5.0% strain levels [24]. All values are taken from that single study. The two amplitudes are used as lower- and higher-strain reference conditions for binder testing and do not correspond to specific pavement structures. Nf, fatigue life; RAR, reacted and activated rubber.

Crumb rubber modification substantially improves LAS-derived fatigue life relative to base bitumen, with the magnitude of improvement scaling with rubber content and, more strongly, with the chemistry of the rubber–asphalt interaction. Terminal-blend modifications alone increased the fatigue life (N_f_) by 50% to 80% compared to unmodified asphalt binder at a 2.5% strain level depending on the rubber particle size, and the addition of wax-based additives (e.g., Sasobit) further extended the fatigue life. However, the enhanced fatigue resistance provided by both the terminal-blend (TB) modification and the wax additives was largely negligible at a higher strain level of 5.0% [12]. Advanced rubber-processing approaches, including devulcanization and RAR modification, have shown substantial potential for improving LAS-predicted fatigue resistance of asphalt binders. It was reported that devulcanized rubber (DVR) modification generally increased fatigue life, with the 9% DVR binder exceeding 5000 cycles at a 2.5% strain level and outperforming the unmodified binder [39]. Binders containing 15–40% RAR exhibited 13–45 times higher fatigue life than the control binder at 5.0% strain, whereas a conventional 12% terminal-blend rubber binder increased fatigue life by approximately 18 times under the same condition [24]. Figure 3 compares the reported LAS fatigue-life ratios of a conventional terminal-blend binder and RAR-modified binders, and shows that the fatigue-life advantage widens sharply as the applied strain decreases, so that a ranking established at one strain amplitude does not carry over to the other. The fatigue-life improvement generally increased with RAR content and was particularly pronounced at the lower strain level, with fatigue-life ratios ranging from 16 to 116 times that of the control at 2.5% strain, compared with 13 to 45 times at 5.0% strain. These ratios are drawn from a single study [24] and represent the highest binder-scale values encountered in this review; the 116-times figure is the upper end of a dose series rather than a typical result.

Beyond comparing the fatigue performance of different binders, the LAS test also provides mechanistic insight into fatigue damage in CRMA binders. Rubberized binders often show delayed peak stress and a slower post-peak decline than neat bitumen, indicating that the dispersed rubber phase helps preserve material integrity over a wider strain range under nonlinear damage conditions that are not fully captured by |G*|sinδ [40]. Furthermore, larger crumb rubber particles (#30–40) produced higher LAS fatigue lives than smaller particles (#60–80) at the same 15% CR content, mainly due to the particle/filler effect [11]. LAS results should nevertheless be extrapolated to mixture performance with caution. Mixture fatigue is also affected by aggregate structure, binder-film thickness, rubber swelling, and rubber–aggregate interaction. The test outcome itself depends on the failure criterion, the strain level, and the analysis procedure, and it does not fully replicate the constant-amplitude cyclic loading experienced in the field [2,13,35].

### 4.3. VECD-Based Fatigue Analysis

VECD theory provides a mechanistic framework for interpreting LAS data and predicting fatigue life as a function of strain, frequency, and temperature. Originating from Schapery’s work-potential theory for elastic and viscoelastic media with evolving damage, VECD represents inelastic behavior through internal state variables governed by thermodynamically consistent evolution laws [41,42]. The framework was adapted for asphalt concrete through the introduction of the pseudo-strain concept, which separates linear viscoelastic time effects from the constitutive response, while distributed damage is represented by an internal state variable linked to the material integrity function [43]. These developments were later formalized in the S-VECD model, where a damage characteristic relationship derived from a single cyclic test is used to predict fatigue life under different stress-controlled or strain-controlled loading conditions, temperatures, and frequencies [44].

A central component of the VECD framework is the damage characteristic curve, which relates material integrity (C) to the accumulated damage parameter (S). As damage progresses, C decreases from its initial value of 1.0, representing the gradual loss of stiffness and structural integrity. At a given level of accumulated damage, a material that retains a higher C value is considered more damage-tolerant. The definition of failure, however, is not common to both material scales. For asphalt mixtures, failure is frequently defined at C = 0.5, corresponding to a 50% reduction in pseudo-secant modulus [43,44]. For asphalt binders, the standard LAS procedure instead defines fatigue life as the number of cycles corresponding to a 35% reduction in the initial value of |G*|sinδ. Criteria based on peak stress, or on the peak in stored pseudo-strain energy, have also been applied to the same data [35,37]. Because these definitions are not equivalent, the criterion adopted should be stated explicitly whenever binder- and mixture-scale VECD results are compared.

For CRMA binders and mixtures, S-VECD and push–pull (PP)-VECD methods have been used to relate binder rheology to mixture-level fatigue performance. Push–pull VECD analysis showed that wet-process, devulcanized, and terminal-blend rubber technologies improved the fatigue resistance of reclaimed asphalt pavement (RAP) mixtures, with wet-process CRMA providing the most favorable overall response [45]. Similarly, S-VECD analysis of wet- and dry-process rubberized stone-matrix asphalt showed strong agreement between binder-level LAS results and mixture-level direct-tension fatigue performance, supporting its use for binder-to-mixture evaluation for CRMA systems [46]. For long-term-aged CRMA containing warm-mix additives, fatigue lives predicted from LAS data using S-VECD analysis showed strong agreement with time-sweep results, confirming the suitability of LAS coupled with S-VECD analysis as an accelerated approach for characterizing binder fatigue damage [2]. VECD also provided a consistent assessment of terminal-blend CR/SBS composite binders and proved more reliable than the phase-angle peak criterion, which showed limited sensitivity to damage progression in highly modified binders [1].

### 4.4. Healing Under Intermittent Loading and Its Representation in VECD

Laboratory fatigue testing applies continuous loading, whereas in-service loading is intermittent. Because asphalt binders recover part of their damaged structure during the intervals between load applications, continuous protocols such as the standard LAS may understate the fatigue life that the same material achieves in the field. For CRMA, this omission is potentially larger than for unmodified binders, since the elastic restoring force of swollen rubber particles contributes to crack closure during unloading, as described in Section 3.2.

The published healing measurements for rubberized systems are summarized in Table 1. At the binder scale, fatigue–healing–fatigue testing of base, SBS-modified and crumb rubber-modified binders across short-, long- and extended long-term aging produced healing indices together with a regression model relating them to rheological parameters [47]; time-sweep testing with short rest periods inserted after a set number of pulses produced a combined fatigue and healing index derived from the ratio of dissipated energy change [48]; fatigue–rest–fatigue testing of pretreated crumb rubber binders produced self-healing rates, which preswelling before microwave irradiation increased by more than 50% [49]; and fatigue–healing–fatigue testing over a wide temperature range produced a regeneration recovery percentage for aged and bio-oil regenerated terminal-blend rubberized asphalt [50]. At the mixture scale, four-point bending of crumb rubber-modified, elastomer-modified and unmodified mixtures under continuous and rest-period loading at 20 and 0 degrees C produced fatigue lives by both stiffness and dissipated-energy criteria [51]; four-point bending of an asphalt rubber mixture with rest periods, following AASHTO T321, produced a model of stiffness ratio as a function of applied strain and rest period from which an endurance limit was derived [52]; and four-point bending of nine modified and two base mixtures found that crumb rubber and SBS-modified mixtures both exhibited pronounced healing [53]. Healing induced externally by microwave heating has also been measured on gap-graded rubberized mixtures in terms of recovered peak load, fracture energy and crack area [54]; this is a different mechanism from the intrinsic recovery under ambient rest considered here and is listed separately.

Taken together, these studies establish that rubberized binders and mixtures recover measurably during rest and that their recovery is at least comparable with that of SBS-modified systems [53]. They do not, however, support a single amplification coefficient for CRMA fatigue life. The quantities reported are not the same quantity: healing indices, dissipated-energy ratios, self-healing rates, regeneration recovery percentages, stiffness-ratio models and recovered fracture energy are defined on different bases and cannot be converted to one another. An amplification coefficient in the sense required would follow from applying continuous and rest-period loading to the same material under otherwise identical conditions, and three of these studies did so, none of them reporting the ratio itself.

The closest available quantity is the healing potential index defined at the binder scale as the relative reduction in the plateau value of the ratio of dissipated energy change when a 10 s rest period is introduced, which its authors relate explicitly to the relative increase in fatigue life produced by that rest, and which was higher for both rubberized binders than for their straight-run base bitumen [48]. It remains an index rather than a life ratio, and it was obtained for three binders at a single rest duration at the binder scale.

At the mixture scale, the corresponding comparison is not presently recoverable. In the one study that applied both loading conditions to the same mixtures, the two protocols were terminated by different rules, without rest at 80% of the initial stiffness or 10^6 cycles, whichever came first, and with rest at a fixed 10^5 cycles, and fatigue lives for specimens that had not reached 50% of initial stiffness were obtained by extrapolating a four-constant double-exponential fit, yielding reported lives that exceed the test duration by more than two orders of magnitude [51]. A ratio formed from those values would therefore reflect the extrapolation rather than a measured difference. The other mixture study derives an endurance limit from a stiffness-ratio model rather than a life ratio [52]. Quantifying the rest-period contribution for CRMA at the mixture scale therefore requires tests in which both loading conditions share a failure criterion and a termination rule; and because the ranking of mixtures has been shown to change with temperature, loading condition and post-processing method [51], any coefficient so obtained would have to be reported together with the temperature, strain amplitude, rest duration and failure criterion at which it was measured.

The mechanism of recovery also differs between rubberized and unmodified binders, which bears directly on how healing should be represented in a damage model. The self-healing capacity of base asphalt correlates with its fluidity, whereas that of both SBS-modified and crumb rubber-modified binders correlates with the elastic recovery ratio [47]. The same separation appears in the temperature domain: elastic recovery leads the healing process in the viscoelastic region, and flow becomes dominant only as the binder enters the viscous flow region [50]. A healing law transferred from unmodified binders, in which recovery is treated as a flow-driven wetting and diffusion process, therefore describes the wrong mechanism for CRMA over much of the temperature range in which fatigue is evaluated.

Two routes are available for incorporating healing into the VECD framework described in Section 4.3, and they differ in what must be measured. The first represents rest as a reduction in the accumulated damage parameter S. Since the damage characteristic curve relating material integrity C to S is treated within the framework as a material property independent of loading history, recovery during rest can be represented as the state point retracing that curve toward lower S, and what is required is an evolution law for S during rest as a function of rest duration, temperature and the damage level at which the rest begins. This route preserves the framework and its single-test calibration, and fatigue–healing–fatigue testing already yields data in the required form, since the modulus on reloading after rest defines the recovered integrity directly. The second route applies an empirical multiplier to the predicted fatigue life, calibrated from tests incorporating representative rest periods. It is simpler, but it is not transferable, because the multiplier is specific to the rest period, temperature and strain amplitude at which it was calibrated and carries no mechanistic content.

For CRMA, the first route requires one further provision. If recovery is governed by the elastic response of the rubber phase rather than by flow of the binder phase [47,50], the rest-period evolution law must be expressed in terms that scale with rubber content and rubber state; and because the rubber network itself changes with aging, as described in Section 5.1, such a law cannot be assumed to remain constant over the service life. No implementation of either route for CRMA was identified in this review. Establishing the first would require fatigue–healing–fatigue testing at several damage levels and rest durations on a material for which the damage characteristic curve has been independently determined, which is achievable with existing equipment and would convert the healing contribution from a qualitative caveat into a term in the damage model.

## 5. Influence of Aging and Crumb Rubber Particle Size on Fatigue Performance

Aging and crumb rubber particle size are among the most important material-level variables controlling the long-term fatigue response of crumb rubber-modified asphalt binders. Aging progressively alters the asphalt matrix through volatilization of light fractions, oxidative hardening, and thermal or UV-induced degradation, while the rubber phase may simultaneously undergo swelling, desulfurization, depolymerization, and release of rubber-derived components into the binder phase [14,15]. Particle size affects both the geometry and reactivity of rubber inclusions, with fine particles facilitating faster rubber–asphalt interaction and medium-to-coarse particles better preserving the elastic inclusion or particle/filler effect [11,55]. Their combined influence determines the relative contribution of rubber–asphalt interaction, elastic energy dissipation, and rubber-derived crack-bridging mechanisms during long-term service [2,11,15].

### 5.1. Effect of Aging on CRMA Binder Fatigue Resistance

Asphalt binder aging is commonly simulated in two laboratory stages. Short-term aging is represented by the rolling thin-film oven test (RTFOT), which reproduces oxidative exposure during mixing and compaction, whereas long-term aging is simulated using the pressure aging vessel (PAV) to represent oxidation during pavement service. During aging, light aromatic and saturated fractions are progressively depleted, while carbonyl and sulfoxide functional groups increase, resulting in reduced molecular mobility and greater binder stiffness [56]. In CRMA binders, these changes occur together with rubber swelling, desulfurization of vulcanized cross-links, and depolymerization of rubber chains, which alter the rubber–asphalt structure and influence fatigue behavior over time [14,15,57].

Fourier-transform infrared spectroscopy (FTIR) indicates that aging affects both the asphalt matrix and the rubber phase. RTFOT and PAV conditioning increase the carbonyl and sulfoxide indices of CRMA binders and are generally accompanied by higher |G*|sinδ values and lower fatigue resistance. Surfactant modification can limit these changes by improving rubber–asphalt compatibility and slowing aging-related deterioration [56]. In high-content CRMA binders, aging also promotes rubber desulfurization and the release of rubber-derived constituents into the bituminous phase, which is consistent with fatigue degradation arising from both asphalt oxidation and changes within the rubber phase [14]. Chemical pretreatment of the rubber itself involves a trade-off: oxidative pretreatment improves rutting resistance but raises carbonyl and sulfoxide indices and increases recoverable creep compliance, which may act against elasticity and fatigue performance [58]. By contrast, modifiers such as amorphous poly-alpha-olefin (APAO) can reduce oxidative aging indices and preserve fatigue performance by stabilizing the rubber–asphalt network [59].

**Figure 4:**
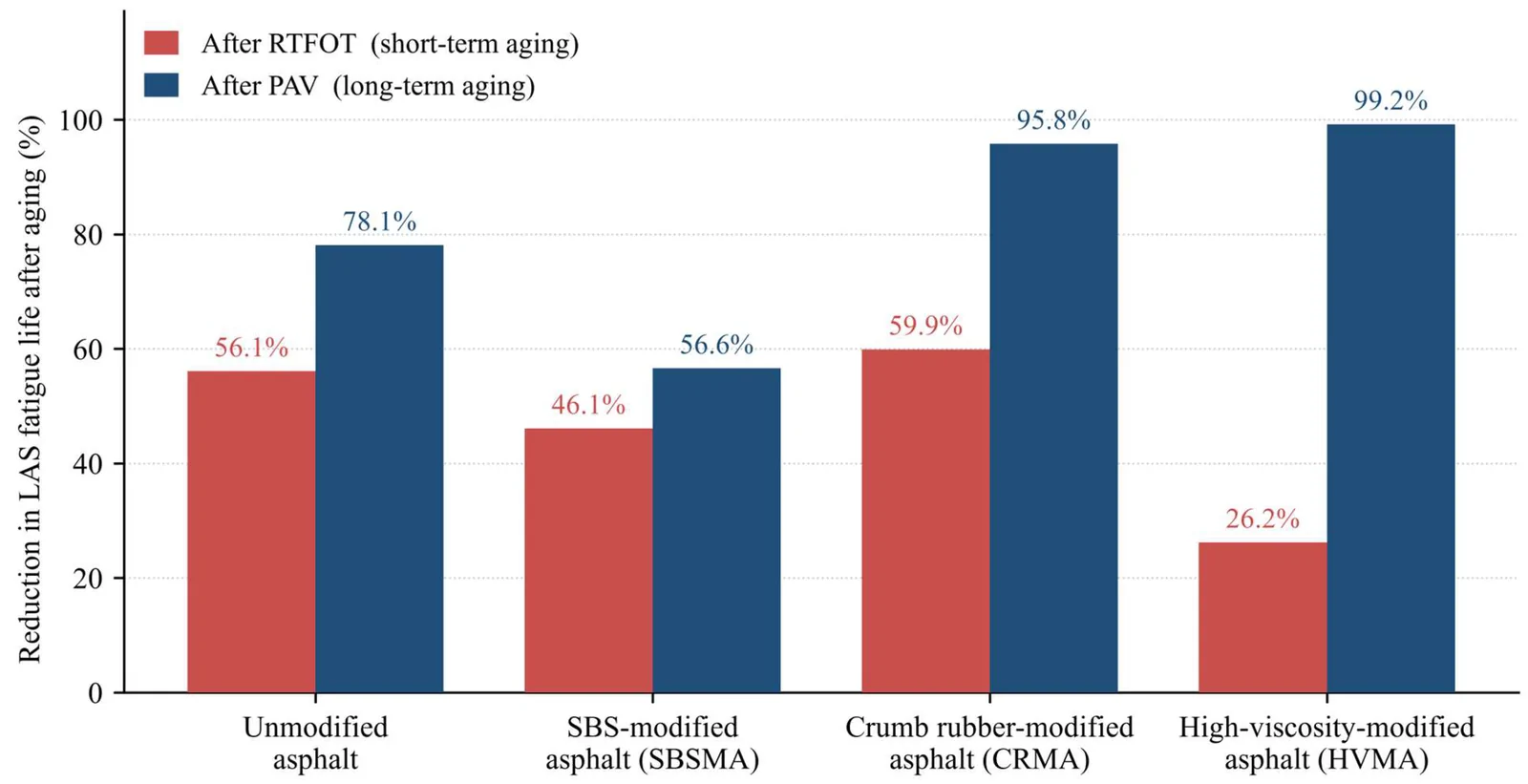
Comparison of LAS fatigue-life reduction after RTFOT and PAV aging for different asphalt binders [60]. The plotted quantity is the change rate of fatigue life, reported as CRNf in that study. RTFOT, rolling thin-film oven test; PAV, pressure aging vessel.

These chemical and structural changes are reflected in damage-based fatigue measurements. The LAS-predicted fatigue life of a pre-manufactured CRMA binder decreased by 59.9% after RTFOT aging and by 95.8% after PAV aging, demonstrating substantial sensitivity to both construction-related and long-term oxidation [60]. Figure 4 compares the corresponding changes in LAS fatigue life among unmodified asphalt, SBS-modified asphalt, CRMA, and high-viscosity-modified asphalt. The loss of fatigue life through aging is substantial for every binder type examined. The relative standing of CRMA after aging therefore depends on how far the control binder has also deteriorated. However, the extent of fatigue deterioration depends on rubber content and preparation method. Terminal-blend high-content CRMA binders retained greater post-aging fatigue resistance than activated crumb rubber systems [14]. Highly cured binders containing 50% rubber showed higher fatigue-aging index values than those containing 30% rubber, while their overall fatigue life decreased less after aging, indicating better retention of fatigue resistance [57]. Aging generally reduces the fatigue life of CRMA binders, although the extent of deterioration depends on the stability of the rubber–asphalt network and the conditions used during binder preparation. Two effects should be separated when this evidence is read. The first is the material change itself, namely oxidative hardening of the asphalt phase and deterioration of the rubber–asphalt interface. The second is the change in the fatigue life predicted by a particular LAS or VECD procedure, which is a property of the procedure as well as of the material. Because aging increases stiffness and LAS-derived fatigue life is strain-dependent, the predicted change can differ in magnitude between low- and high-strain amplitudes for the same aged binder. Reported aging effects should therefore be attributed to the procedure, failure criterion and strain amplitude under which they were obtained rather than read as a single material property.

Although aging reduces the absolute fatigue life of CRMA binders, the residual rubber-derived elastic network may allow them to retain greater fatigue resistance than neat bitumen after PAV conditioning [2,61]. After extended PAV aging, the LAS fatigue life of the oil–rubber composite-modified asphalt was 269% higher than that of the unmodified VG30 binder. In comparison, rubber-modified asphalt and oil-modified asphalt exhibited increases of 84% and 106%, respectively [62]. These results suggest that the post-aging fatigue response depends not only on the presence of crumb rubber but also on the stability and composition of the modified binder network. The direction of this effect is not uniform across studies. In a comparison of PAV-aged binders tested by LAS at 25 °C and at 2% and 4% strain, a crumb rubber-modified binder and a trans-polyoctenamer-activated crumb rubber-modified binder both gave shorter fatigue lives than the base bitumen, while an SBS-modified binder gave the longest [63]. Evidence of this kind is consistent with the separation made above: whether aging is judged to improve or to reduce fatigue resistance depends on the procedure, the strain amplitude and the comparator against which the aged binder is placed.

### 5.2. Improved Laboratory Aging Protocols and Their Adequacy for Two-Phase Aging

The limitations of the standard RTFOT-PAV sequence have prompted several alternative conditioning protocols. These fall into three families: increased thermal severity, simplified thin-film thermal conditioning, and ultraviolet (UV)-coupled conditioning.

The first family retains the PAV principle and raises severity. The premise is that the current protocol ages binders less severely than field exposure, and extensions of the 20 h conditioning period to 40 or 60 h have been proposed on that basis [64]. The second family replaces the pressure vessel with thin films in a forced-air oven. For long-term conditioning, a simplified Universal Simple Aging Test gave high correlations with the 20 h PAV for stiffness, relaxation rate, the fatigue factor and the Glover–Rowe parameter across four neat binders, two asphalt–rubbers and two polymer-modified binders, with the 40 h conditioning time identified as the main obstacle to implementation [65]. The corresponding short-term protocol agreed with the RTFOT for MSCR and LAS responses on the same material set, and assigned the same performance grades and traffic levels [66]. Alternative procedures covering both stages have also been proposed for agencies without PAV equipment [67]. These are the only improved thermal protocols among those reviewed here that have been evaluated on asphalt–rubber binders alongside polymer-modified and unmodified binders.

The third family couples UV exposure with thermal conditioning. On a set of twelve binders including a ground tire rubber-modified binder, a UV route and a thermal route with varying exposure times, both reached the critical block-cracking zone in Glover–Rowe terms, but the oxidative effect characteristic of field exposure was more evident in the UV route [64]. UV conditioning has been applied to crumb rubber-modified systems at both scales: surface cracking appeared later and developed more slowly in rubber-modified asphalt than in the unmodified control, and the increase in large molecular weight was smaller and delayed, indicating that the rubber phase retards UV aging [68]. Under filtered xenon-arc exposure for 10 to 30 days, crumb rubber ranked below SBS but above ethylene vinyl acetate in retained cracking resistance, which chemical analysis attributed to the lower oxidation resistance of the rubber network relative to the three-dimensional network formed by SBS [69]. Because UV absorption is confined to a surface layer, the outcome depends strongly on specimen geometry: under variable-intensity UV, rubber-modified asphalt reached comparable high-temperature performance and elastic recovery in 1.5 mm films after three cycles and in 2.0 mm films after six, and in 1.0 mm films after six cycles and in 1.5 mm films after nine, so that film thickness and radiation dose are interchangeable within limits for those properties [70]. One study in this family addressed the rubber–asphalt interface directly. Digital image correlation applied to single-edge-notch bending of crumb rubber-modified asphalt mortar showed that the interfacial crack growth rate increased progressively with UV exposure time, and an interface damage factor derived from the horizontal strain field tracked the accompanying reduction in interfacial bond strength [71]. This is the only conditioning study reviewed here in which the measured response is a property of the interface rather than of the bulk binder.

Which family is better suited to simulating two-phase aging in CRMA cannot be decided from the evidence as presently reported, and the reason is instructive. All of these protocols have been validated by correlation of bulk rheological responses with those of a reference laboratory procedure. Such a correlation establishes that a protocol brings the asphalt phase to a comparable aged state; it does not establish that the rubber phase has been brought to a comparable state, because the two phases are driven differently. Oxidation of the asphalt phase is accelerated by temperature and oxygen availability, whereas desulfurization of vulcanized cross-links and depolymerization of rubber chains are governed primarily by temperature and time [14,15,57]. Raising thermal severity to compensate for insufficient oxidation therefore does not act neutrally on the rubber phase, and a protocol calibrated to reproduce the field oxidation state of the asphalt phase may simultaneously overstate rubber degradation. That degradation is measurable on its own terms: the degree of desulfurization of crumb rubber, expressed through the sol fraction and the decrosslinking degree, correlates with the fracture energy density of the resulting binder, and beyond a threshold sol fraction, the integrity of the rubber network is compromised, and intermediate-temperature cracking resistance deteriorates [72]. UV-coupled conditioning reproduces the oxidative signature of field exposure more closely at lower bulk temperature and is the only family that has so far been used to measure an interfacial response, but its action is confined to a surface layer, and its results are therefore geometry-dependent in a way that pressure-vessel conditioning is not.

On this evidence, the more useful conclusion concerns the validation target rather than the protocol. A conditioning procedure intended for CRMA should be validated against phase-resolved indicators, carbonyl and sulfoxide indices for the asphalt phase together with measures of change in the rubber network, such as the release of rubber-derived constituents, the sol fraction, or the interfacial damage measures described above, rather than against bulk rheological equivalence with a reference thermal procedure. Combined thermal and UV conditioning, with film thickness fixed and severity calibrated against field cores rather than against a laboratory reference, is the configuration most consistent with the two-phase mechanism described in Section 5.1. Such a configuration has not yet been implemented or validated for CRMA, and field-aged reference material for the calibration remains scarce; one recent study conditioned rubberized mixtures containing reclaimed asphalt pavement under authentic field exposure for up to nine months and proposed aging indices for the resulting viscoelastoplastic change [73].

### 5.3. Influence of Crumb Rubber Particle Size on Fatigue Performance

Particle size controls both the surface area available for rubber–asphalt interaction and the extent to which rubber remains as discrete inclusions within the binder [74]. Its influence on fatigue resistance is therefore associated with two complementary mechanisms: a particle or filler effect, in which undissolved rubber particles provide elastic reinforcement, and an interaction effect, in which swelling, depolymerization, and material exchange at the rubber–asphalt interface modify the continuous binder phase [11,74]. Size classification conventions, the conversion between mesh number and particle diameter, and the synthesis of particle-size effects on rheology and storage stability are set out in a companion review by the present authors [75]; this section is confined to fatigue.

In wet-process CRMA binders, larger particles may provide greater LAS fatigue life by preserving a stronger elastic inclusion effect under cyclic loading [11]. In contrast, finer particles interact more rapidly with asphalt because of their higher surface-to-volume ratio, promoting swelling, devulcanization, and the release of rubber-derived polymer chains and oils into the binder phase [74]. This greater interaction may improve fatigue resistance when the released components are effectively incorporated into the asphalt matrix. However, extended thermal conditioning can weaken the particle-size benefit because asphalt oxidation and continued rubber degradation progressively reduce LAS fatigue life in both fine and coarse particle systems [11,74].

Experimental findings further show that the preferred particle size depends on the CRMA production method. In bio-oil-pretreated binders, predicted fatigue life increased with increasing mesh number, so that the finer fractions gave the longer fatigue lives, and the fatigue resistance parameter was highest for the 100 mesh (0.15 mm) fraction [76]. The reported correlation is expressed in terms of mesh number rather than particle diameter, which is why a size-based reading of that study can suggest the opposite trend [76]. An opposite trend has been reported for CR/SBS composite binders, in which larger crumb rubber particles enhanced rutting and fatigue resistance [55]. The divergence between these two systems is consistent with the production-method dependence noted above, because rapid crack propagation has been associated with the vicinity of un-depolymerized rubber, and coarser particles require longer times and higher temperatures to depolymerize [76]. For high-content CRMA binders, 60-mesh activated rubber provided a favorable balance between fatigue life and material stability [77].

The particle-size relationship may be reversed in terminal-blend systems. High-temperature and high-shear processing promote extensive rubber dissolution, reducing the contribution of discrete particles and making rubber–asphalt interaction the dominant modification mechanism. Under these conditions, finer particles dissolve more completely and release a greater concentration of active polymer fragments, resulting in greater fatigue improvement than partially undissolved coarser particles [12]. Particle-size effects at the mixture-level may also differ from binder-level trends because aggregate structure, binder-film thickness, adhesion, and rubber distribution become influential. CRMA mixtures containing finer rubber particles, particularly 80 mesh, exhibited longer fatigue life and lower crack-growth rates than mixtures containing coarser rubber. This improvement was attributed to the easier incorporation of fine particles into the binder film, which promoted a more uniform distribution of elastic inclusions and reduced local stress concentrations near the aggregate–binder interface [21]. Overall, no single particle size can be considered universally optimal. Its effect on fatigue resistance depends on whether particle reinforcement or rubber–asphalt interaction dominates, which is controlled by the processing method, material scale, and degree of rubber dispersion.

## 6. Comparison of Wet and Dry Processes in Crumb Rubber Asphalt Fatigue Performance

The method of crumb rubber incorporation determines whether fatigue resistance is governed primarily by the particle effect or by rubber–asphalt interaction. In the wet process, crumb rubber is blended with the binder at high temperature, promoting particle swelling, absorption of lighter fractions, and development of an elastic binder network that enhances recoverable deformation. In the dry process, crumb rubber is added to the heated aggregates, commonly as a partial replacement for fine aggregate, and the limited interaction time causes the rubber to contribute mainly through its particle-level elastic response. Dry-process fatigue performance therefore depends more strongly on rubber dosage, particle function, and digestion conditions than wet-process performance, in which the rubber is incorporated into the binder phase before mixing [4]. Incorporation into the binder phase has itself been shown to raise binder fatigue resistance [18]. The wet, dry and semi-wet comparisons assembled in this section are drawn from different studies rather than from a common experimental program, and rubber content, particle size, digestion time and mixing temperature are not held constant across them. These variables are confounded with the incorporation method, so the comparison indicates the range of reported behavior rather than an isolated process effect. A standardized comparison would require the digestion time, the rubber particle size and the dosage basis to be fixed. The dosage basis in particular should be stated, because rubber expressed as a percentage of binder mass and rubber expressed as a percentage of aggregate mass are not interchangeable quantities. One comparison avoids this confounding by design. Four gap-graded surface mixtures with the same mineral skeleton were tested under repeated loading, differing only in the binder: a conventional bitumen, an SBS-modified bitumen, a high-viscosity rubber-modified bitumen produced by the wet process, and the same conventional bitumen with crumb rubber added by the dry process. The three modified mixtures had similar cracking lives and all exceeded the conventional mixture, with no significant difference between the wet and dry routes [78]. In that study, the rubber-modified mixtures delayed crack initiation but propagated damage more rapidly once cracking had begun, and the dry-process dosage was expressed as a percentage of total mixture mass, which illustrates the reporting problem noted above.

Although the dry process is commonly considered particle-dominated, recovered-binder studies show that it can also produce modification of the asphalt phase. Binder extracted from dry-process mixtures exhibited greater elasticity and lower stress sensitivity than the corresponding base binder, while DSR and multiple stress creep and recovery (MSCR) results indicated that approximately 57–62% of the added rubber had interacted with the asphalt [79]. Thus, wet and dry processing differ primarily in the extent and control of rubber–asphalt interaction. For dry-process mixtures, a conditioning period of approximately 90 min was reported to promote sufficient interaction, whereas plant mixing temperatures above 190 °C reduced the effectiveness of crumb rubber and lowered fatigue resistance [80,81].

When sufficient rubber–asphalt interaction is achieved, dry-process mixtures can exhibit fatigue performance comparable to that of wet-process mixtures. For stone-matrix asphalt containing 10% crumb rubber by binder mass, direct-tension S-VECD testing showed that the dry-process mixture had a fatigue life similar to, or slightly greater than, that of the wet-process mixture. The corresponding binder-level LAS results produced the same performance ranking, supporting the use of binder testing to compare fatigue behavior between the two incorporation methods [46,82]. Similarly, gap-graded dry-process asphalt rubber exhibited fatigue resistance comparable to wet-process asphalt rubber when fine crumb rubber and adequate interaction time were used [80]. A comparison of the wet, dry, and semi-wet routes in gap-graded mixtures found that the semi-wet route matched or exceeded the wet process after only 30 min of digestion, whereas the dry-process mixture remained inferior at the same digestion time [83]. The durability of this equivalence has also been observed in service. A gap-graded dry-process mixture containing 20% crumb rubber by binder mass sustained approximately 1.2 million equivalent single-axle loads (ESALs) over eight years on a low-to-medium-volume road, and retained the fatigue tendency established for wet-process mixtures in companion studies [84]. Furthermore, an optimized dry-process mixture containing ultrafine crumb rubber achieved at least ten times the cracking life of a conventional mixture of equivalent thickness [85]. As this result was obtained from a reflective-cracking assessment rather than a repeated-load fatigue test, it is treated here as supporting rather than direct fatigue evidence.

A contrasting result was observed in dense-graded mixtures. The high-viscosity wet-process mixture containing 25% crumb rubber by binder mass exhibited the highest fatigue resistance. In contrast, the dry-process mixture containing 41% crumb rubber as an aggregate substitute showed lower fatigue resistance and weak internal cohesion. Although the dry-process mixture still outperformed the unmodified 50/70 mixture, this benefit was attributed largely to its relatively high asphalt binder content (6.875% of the total mixture mass). These findings indicate that controlled binder-phase modification favors fatigue performance, whereas excessive rubber use as an aggregate substitute can compromise the structural integrity of dry-process mixtures [86]. Similarly, supporting dry-process studies showed that moderate crumb rubber contents combined with adequate curing preserved fatigue resistance, whereas higher contents reduced fatigue life unless the rubber–binder interface was improved [87,88]. These findings have encouraged the development of intermediate technologies that combine the controlled rubber–asphalt interaction of wet processing with the production simplicity of dry incorporation. Activated rubber pellets (ARP) improved the cracking resistance of mixtures containing 30% RAP [89], while pre-swollen crumb rubber promoted a more ductile fracture response [90]. A hybrid wet–dry method also produced the highest cracking tolerance index among the evaluated incorporation routes, although this index was obtained from a monotonic rather than repeated-load fatigue test [91]. The comparative fatigue and cracking outcomes reported for wet, dry, and intermediate crumb rubber incorporation routes are summarized in Table 2.

## 7. Field Fatigue Performance and Mechanistic Modeling

Although binder- and mixture-scale studies generally show improved fatigue resistance with crumb rubber modification, laboratory performance cannot be directly translated to pavement behavior because field cracking is influenced by traffic, climate, aging, and construction variability. Field fatigue performance should therefore be evaluated using observed distress in full-scale sections, testing of field-recovered cores, and mechanistic–empirical predictions supported by laboratory data.

### 7.1. Field Fatigue Performance

Among the available full-scale studies, the National Center for Asphalt Technology (NCAT) Pavement Test Track provides strong evidence of the cracking resistance of CRMA. A dense-graded wet-process mixture containing 11% ground tire rubber carried 20 million ESALs over two research cycles without cracking, performing comparably to an SBS-modified section. Gap-graded asphalt–rubber sections also showed high cracking resistance, with no cracked lane area after 16 million ESALs, while the 20% RAP control increased from 11% to 45% cracking. In a separate structural evaluation, a CRMA section endured 17% more load repetitions than its control before reaching the cracking threshold [93]. Public road monitoring provides additional evidence of the field cracking performance of CRMA asphalt. Across five Louisiana projects in service for more than eight years, wet- and dry-process CRMA sections exhibited statistically comparable or better resistance to alligator and transverse cracking, as well as better overall pavement condition, than companion control sections, although SBS-modified controls outperformed CRMA in some cases [94]. Similarly, after five years of service in Michigan, transverse cracks in the control lanes terminated at the boundaries of adjacent lanes containing pre-swollen crumb rubber, indicating improved resistance to crack propagation under field conditions [90].

However, field-core evidence provides a more balanced assessment of the long-term fatigue performance of CRMA relative to SBS modification. Cores collected over 63 months from three heavily trafficked Spanish highways showed comparable stiffening and fatigue evolution in CRMA and SBS surface courses, with both becoming progressively more susceptible to fatigue damage after an initial strain-hardening period of 20 to 40 months [95]. Comparisons between laboratory and field performance showed that conditioning at 135 °C for nine days was required to replicate the stiffness increase and fatigue deterioration observed in long-term field cores, highlighting the strong aging–fatigue interaction and the limitations of short-term aging protocols for predicting long-term field performance [96]. Laboratory fatigue testing of mixtures taken from a field project provides a further comparison against a polymer-modified control. Beam specimens of the reference, polymer-modified and rubber-modified gap-graded mixtures placed on a highway section in Stockholm were tested in strain-controlled four-point bending, and the three mixtures were found to differ statistically, the rubber-modified mixture giving the longest fatigue life, followed by the polymer-modified and then the reference mixture [97]. That study also reports fatigue-life ratios between the three mixtures; because such ratios depend on the strain amplitude at which the fitted relationships are compared, only the ranking and the statistical outcome are carried forward here. The available field and field-core evidence on CRMA fatigue and cracking performance is summarized in Table 3.

### 7.2. Mechanistic Modeling of Fatigue Performance

The limited availability of long-term field data has increased the importance of mechanistic models for translating binder- and mixture-scale fatigue properties into pavement-level performance predictions. Mechanistic fatigue models have been developed at the binder, mixture, and pavement scales, ranging from crack-growth and continuum-damage formulations to structural mechanistic–empirical simulations. At the binder scale, a damage-mechanics-based DSR-C-model combined with a pseudo-J-integral Paris law was used to predict circumferential crack growth during time-sweep testing. The Paris-law coefficients were largely independent of loading frequency and strain amplitude, while crack growth increased with aging and decreasing temperature [98]. The modified Paris-law approach was subsequently applied to characterize fatigue crack-density evolution in SBS/CR composite binders containing 30–40% CR [17]. Similarly, LAS-derived S-VECD predictions indicated that fatigue life increased with CR content up to 20% in Sasobit- and Zycotherm-modified warm-mix binders [99].

At the mixture scale, an integrated model combined a generalized Maxwell formulation for time-dependent viscoelastic behavior, an overstress component for permanent deformation, and S-VECD theory for fatigue damage through progressive pseudo-stiffness loss, with failure defined at a 50% reduction. The model was calibrated using indirect tensile creep, Marshall, and cyclic indirect tensile (IDT) fatigue tests. It reproduced optimum fatigue performance at 10–15% CR by binder mass and accelerated deterioration at contents of 20% or higher, which shows that it captures the nonlinear effect of CR dosage on fatigue behavior [100]. Beyond cyclic fatigue models, fracture-mechanics studies provide supplementary evidence on the crack-retarding effect of crumb rubber. R-curve and mixed-mode fracture analyses showed that mixtures containing 20% CR exhibited greater resistance to crack propagation and a delayed transition from quasi-brittle to brittle fracture [101,102].

At the pavement scale, fatigue relationships derived from four-point bending tests are commonly used in structural design to estimate allowable load repetitions from tensile strain. Using these relationships, asphalt–rubber mixtures were predicted to achieve the same design life as conventional mixtures with at least a 50% reduction in surface-layer thickness. A companion finite-element analysis also predicted reflective-cracking lives nearly eight times greater than those of conventional overlays [103,104]. Mechanistic empirical analysis using PP-VECD and AASHTOWare Pavement ME predicted that recycled tire rubber-modified mixtures could provide fatigue lives three to fifteen times longer than PG58-28 or PG58-34 mixtures, depending on pavement structure and mode of cracking. For thick-pavement top-down cracking, the predicted maintenance-initiation time increased from 10.5 months for PG58-34 to 154.8 months for the crumb rubber terminal-blend mixture [41].

Complementary modeling studies reported similarly favorable fatigue performance. A 3D-Move analysis predicted fatigue lives five to seven times longer than those of an identical pavement containing unmodified asphalt [105]. Damage mechanics modeling extended the predicted crack-initiation life from 5.4 to 7.6 years [106]. For pavement reconstruction using a dry-process CRMA mixture, Pavement ME projected lower bottom-up fatigue cracking over a 20-year design period, consistent with laboratory fracture-energy improvements of 29–50% [107]. Table 4 summarizes the mechanistic modeling approaches used to assess the fatigue performance of CRMA at the binder, mixture, and pavement scales. Read together, the entries show that the predicted benefit depends as much on the transfer function and calibration data adopted as on the material itself, which is why these predictions are treated here as comparative rather than absolute.

## 8. Discussion

### 8.1. Factors Influencing CRMA Fatigue Performance

The fatigue resistance of CRMA is generally interpreted in terms of the combined contribution of rubber–asphalt interaction and the elastic response of undissolved crumb rubber particles [9,10,11]. However, the particle effect should not be interpreted as uniformly beneficial because increasing rubber content does not always improve fatigue resistance. The available evidence indicates that the contribution of undissolved rubber depends strongly on the condition of the rubber–asphalt interface. When particles are adequately swollen, dispersed, and well-bonded, they can store and recover strain energy, slow crack propagation, and promote more ductile post-peak behavior [19,26,108]. In contrast, inadequate mixing or prolonged aging can weaken the bond between crumb rubber and the asphalt binder, creating localized stress concentrations that promote damage initiation [28,74].

Reported optimal crumb rubber contents vary considerably among studies because the observed fatigue response depends on the damage stage evaluated, material scale, dosage basis, and processing method [17,28,86,100]. A rubber content that delays crack initiation at the binder scale may not provide the same benefit during crack propagation or mixture-level testing. Binder specimens also exclude the effects of aggregate structure and mixture cohesion, which may result in a higher apparent optimal dosage than that observed in asphalt mixtures. In addition, rubber used as an aggregate substitute cannot be directly compared with rubber used as a binder modifier because it performs a different function within the material. Particle-size effects are similarly influenced by the processing method and evaluation scale. Coarser particles may provide better fatigue performance when wet processing preserves their elastic contribution, whereas finer particles may be more effective in terminal-blend systems because they dissolve and interact more readily with the asphalt binder [11,12]. At the mixture scale, finer particles may also improve fatigue resistance by achieving a more uniform distribution within the binder film [21]. Consequently, no single particle size can be considered universally optimal; its effectiveness depends on the degree of particle survival, rubber–asphalt interaction, and dispersion within the mixture.

In addition to material composition and processing conditions, the measured fatigue benefit of crumb rubber modification is strongly influenced by strain amplitude. Strain level should therefore be reported explicitly when interpreting LAS results rather than being treated solely as a limitation of the test protocol. Across LAS studies, the relative fatigue benefit of CRMA was generally greater at 2.5% than at 5.0% strain, although highly modified systems retained substantial improvement at the higher strain amplitude [12,24]. This strain dependence indicates that fatigue performance should not be interpreted from a single LAS fatigue-life value without considering the applied strain level. Because 2.5% and 5.0% strain are commonly used as lower- and higher-strain reference conditions in LAS testing, fatigue life should be evaluated at both amplitudes and interpreted in relation to the expected strain level [39]. The contrasting findings on crumb rubber dosage, particle size, and strain amplitude are synthesized in Table 5, which highlights the key factors contributing to differences among the reviewed studies.

Aging generally reduces the fatigue life of CRMA, although aged CRMA may still exhibit greater fatigue resistance than the corresponding aged unmodified binder. This relative advantage does not necessarily indicate superior aging resistance, as it may partly reflect greater deterioration of the control binder. Field-core studies further indicate that conventional laboratory aging procedures may not fully reproduce the long-term aging experienced under in-service pavement conditions [60,62,96]. Therefore, aging performance should be assessed using both absolute changes in fatigue life and comparisons with aged control materials, while laboratory findings should be validated against field-recovered binders and mixtures.

### 8.2. Evaluation of CRMA Fatigue Testing, Modeling, and Field Performance

Across the available studies, |G*|sinδ and the Glover–Rowe parameter can serve as screening indicators, but their basis in linear rheological response limits their ability to characterize progressive fatigue damage in highly modified CRMA binders [35,109]. For |G*|sinδ, weak correlations with measured fatigue life and inconsistent fatigue rankings have been reported for aged and highly modified binders [2,13,14,16]. These findings support the use of damage-based methods for a more comprehensive assessment of fatigue performance. A comparison carried out on a single set of materials makes the same point in a more direct way. When a crumb rubber-modified binder and three warm-mix variants of it were evaluated by |G*|sinδ, LAS, a mortar shear fatigue test, indirect tensile fatigue testing, and four-point bending, the LAS, mortar, and bending results agreed on the ranking of the materials, whereas |G*|sinδ and the indirect tensile fatigue results did not [110]. Disagreement therefore arises not only between damage-based and linear viscoelastic indicators but also between damage-based tests of different specimen geometries.

LAS/VECD approaches generally provide more consistent fatigue rankings than linear viscoelastic indicators, although fatigue-life predictions remain sensitive to the selected failure criterion. LAS-derived fatigue predictions have shown good agreement with time-sweep results for long-term-aged CRMA and have provided a more consistent fatigue assessment than the phase-angle-peak criterion in highly modified binders [1,2]. However, the interpretation of LAS results is influenced by two methodological factors. The first is the failure criterion used to determine fatigue life, which can substantially affect the calculated fatigue life of highly elastic rubber-modified binders. A fixed reduction in material integrity may identify failure while the binder still retains part of its load-carrying capacity, resulting in a lower estimated fatigue life than an energy-based criterion such as the peak in stored pseudo-strain energy [2,111]. Therefore, LAS-derived fatigue lives should be compared across studies only when the analysis procedure and failure criterion are explicitly reported. The second is the translation of binder-level results to mixture performance. Binder-level LAS results have shown good consistency with mixture-level S-VECD rankings when the modified binder phase largely controls the fatigue response [46,82]. However, this consistency may decrease when crumb rubber remains as a discrete component within the mixture and contributes directly to the overall fatigue response [86]. Monotonic fracture tests can provide useful information on cracking tolerance, but they do not represent repeated-load fatigue behavior and should therefore be interpreted separately from fatigue-life rankings [91]. The control mode under which a test is run has a comparable effect on what the result means. In stress-controlled indirect tensile fatigue testing of stone-matrix asphalt containing up to 20% crumb rubber, fatigue life increased with rubber content, and the improvement was larger at the higher applied stress levels, and an optimum rubber content was reported beyond which fatigue life decreased [112]. Because a stiffer mixture carries a smaller strain under the same applied load, an increase in stiffness lengthens fatigue life in a stress-controlled test while shortening it in a strain-controlled test, so fatigue lives obtained under the two modes should not be compared directly.

Across the reviewed studies, the largest reported fatigue improvements of CRMA occur at the binder scale and the smallest at the field scale, as summarized in Table 6. The pattern is better described as a narrowing of the range of reported values than as a monotonic attenuation, because the results at each scale overlap and include exceptions in both directions. At the binder scale, the reported outcomes extend from fatigue lives shorter than that of the base bitumen, in a comparison of PAV-aged binders evaluated by LAS at 25 °C in which an SBS-modified binder ranked highest [63], to approximately 116 times the control at 2.5% strain [24]. The mixture scale shows the same overlap. A study that fixed the mineral skeleton and varied only the binder found rubber-modified and SBS-modified mixtures to have similar cracking lives, both longer than that of the conventional mixture [78]. Four-point bending of mixtures taken from a field project, by contrast, ranked the rubber-modified mixture above both the polymer-modified and the conventional mixture [97]. The Table is arranged so that the reported gain can be read against the evidence tier and the comparator applied at each scale. Binder-level LAS studies report a wide range of fatigue responses. Terminal-blend binders in one study showed values close to those of the control at 5.0% strain [12], whereas reacted and activated rubber systems reached approximately 116 times the control at 2.5% strain and 45 times at 5.0% strain [24]. At the mixture-scale, the reported improvements are generally more moderate, ranging from approximately 1.4 to 10 times those of the corresponding control mixtures [28,46,100], with further supporting evidence from a reflective-cracking assessment [85]. Several mixture-scale studies report the comparison as a ranking against a named control rather than as a ratio and therefore do not enter this range [78,97,112]. The upper bound of this mixture-scale range has not been confirmed against a repeated-load fatigue result and is therefore treated as provisional throughout, as noted in Table 6. Across the reviewed modeling studies, mechanistic models predict fatigue-life improvements of approximately 1.4 to 15 times relative to the corresponding control structures, including an increase in maintenance-initiation time from 10.5 to 154.8 months [41,103,104,105]. In contrast, field studies generally indicate performance that is comparable to or moderately better than that of modified control sections [93,94,95].

The differences between laboratory and field-scale fatigue performance should be attributed to three distinct influences, which are not separable in the present evidence base and are therefore reported together. The first is a scale effect: moving from binder to mixture and pavement evaluation introduces aggregate structure, mixture cohesion, construction variability, environmental exposure, and aging. The second is a comparator effect: binder studies commonly compare CRMA with unmodified binders, whereas field studies commonly use SBS-modified or well-designed conventional pavements as controls, so the reference material becomes stronger as the evaluation scale increases. The third is a method effect: the tests applied at each scale differ in loading mode, failure definition, and damage range, so the reported ratios are not measured on a common basis. Because no reviewed study holds two of these three influences constant while varying the third, the differences observed between scales cannot presently be assigned to scale alone. The discussion below separates the first two as far as the available evidence permits. First, moving from binder-level testing to mixture and field evaluation introduces additional influences, including aggregate structure, mixture cohesion, construction variability, environmental exposure, and aging, that are not represented in binder tests. Second, the reference materials used for comparison also differ across evaluation scales. Binder studies commonly compare CRMA with unmodified binders, whereas field studies often use SBS-modified or well-designed conventional pavements as controls. Consequently, the smaller improvements reported at the field scale may reflect both the additional factors affecting pavement performance and the use of stronger control materials. When CRMA is compared with a less fatigue-resistant control, however, substantial improvements may still be observed. Overall, the available field evidence is consistent with CRMA performing at a level comparable to polymer-modified asphalt under the conditions examined. It does not demonstrate consistent superiority, and because of the comparator effect described above, equivalence at the field scale should not be read as evidence of a smaller material benefit. Direct comparisons against polymer-modified controls do not point in a single direction: a rubber-modified mixture ranked above a polymer-modified mixture in four-point bending [97], mixtures sharing the same mineral skeleton gave similar cracking lives for the two modifications [78], and a comparison of PAV-aged binders placed the SBS-modified binder ahead of the rubber-modified one [63]. Taken together, these results support equivalence as a general observation across the conditions examined rather than as a ranking that holds at every scale.

The magnitude of the comparator and method effects can be bounded even though they cannot be separated from the scale effect. Within a single study using one control binder, the reported improvement fell from about 116 times to 45 times when the strain amplitude was raised from 2.5% to 5.0% [24], so a change in test condition alone moves the ratio by a factor of about 2.6. A change in comparator moves it by a comparable amount, as the three studies cited above show. The decomposition nevertheless cannot be completed at the field scale, because none of the field studies reviewed here can be confirmed to use an unmodified comparator: one names a polymer-modified comparator [95], one refers only to corresponding control sections without identifying the material [94], and for the third no abstract could be obtained [93]. We therefore do not estimate what the field ratio would be against a common unmodified control, and the field row of Table 6 should be read as conditional on a polymer-modified or well-designed conventional reference.

The dependence of the reported fatigue life on the failure criterion is not a marginal effect, and the studies in which more than one criterion, or more than one fatigue approach, was applied to the same data set are collected in Table 7. Eight such studies were identified among those reviewed. Two properties of this evidence should be stated before the Table is read. First, none of these studies report a numerical ratio between the fatigue lives obtained under different criteria for the same material; what they report is whether the ranking of materials is preserved. A summary of ratios, in the form that would be most useful, cannot therefore be assembled from the present literature, and the Table reports the comparison actually made in each study. Second, the disagreements are not confined to the contrast between linear viscoelastic indicators and damage-based analysis. They also occur between damage-based tests of different specimen geometries, and between failure criteria applied within a single test.

The clearest case specifically concerns rubber-modified binders. Five LAS failure criteria applied to unmodified, polymer-modified and asphalt rubber binders, and to the corresponding mixtures, under several aging and temperature conditions, showed that the standard criterion based on the peak in stored pseudo-strain energy frequently failed to represent the improvement that the mixtures displayed, whereas criteria based on maximum shear stress and on the integrity curve at 25 °C produced the binder fatigue factor most strongly correlated with the mixture fatigue factor [113]. A comparable result is reported for a hybrid binder containing crumb rubber, where the 35% reduction criterion under-rated the fatigue performance that an energy-based criterion resolved [111]. The direction is consistent: criteria that declare failure at a fixed loss of integrity penalize highly elastic rubber-modified binders, which retain load-carrying capacity beyond that point. Within a single time-sweep data set on a terminal-blend crumb rubber and SBS composite binder, four criteria and two damage models were compared, and the phase-angle-peak criterion was found unsuitable for that material while energy-deviation and VECD analyses agreed most closely with the observed cracking [1].

The choice of criterion also governs whether a result transfers between test conditions. Within the VECD framework, a comparison of the G^R and W_sum^R criteria on crumb rubber, waste cooking oil and SBS-modified binders found that both criteria agreed that rubber and polymer modification improve fatigue resistance, but that G^R remained temperature-dependent whereas W_sum^R unified results across both test methods and all temperatures [114]. Agreement on ranking at one temperature therefore does not imply agreement across a test program. Where the comparison is made across test configurations rather than criteria, the same pattern appears: five approaches applied to one set of warm asphalt rubber materials agreed on the ranking for the linear amplitude sweep, mortar shear fatigue and four-point bending, and disagreed for the Superpave fatigue parameter and the indirect tensile fatigue test [110]; four failure criteria applied across time sweep, LAS and elastic recovery testing of seven modified binders, including crumb rubber and terminal-blend systems, preserved the advantage of the elastomeric binders while showing that LAS fatigue life correlates with elastic recovery, increasingly so at higher strain [115]; and for crumb rubber binders evaluated under both stress-controlled and strain-controlled time sweep loading, the stress- or strain-to-failure relationship itself depends on the control mode, so that criterion and control mode cannot be varied independently [116]. The practical consequence is that a reported CRMA fatigue life is interpretable only together with the criterion, the test configuration, and the control mode under which it was obtained, and that rankings reported under one criterion should not be carried across to another.

Mechanistic models provide a useful link between laboratory testing and pavement-scale performance, but their predictions remain strongly dependent on laboratory inputs and transfer functions developed mainly for conventional dense-graded mixtures. In addition, current models do not consistently account for factors such as rubber morphology, healing, and the interaction between aging and fatigue damage. Their results are therefore more appropriate for comparing materials and structural scenarios than for directly estimating long-term pavement service life.

## 9. Limitations of CRMA Binder Fatigue Cracking Evidence and Research Needs

Despite advances in fatigue evaluation, the interpretation of CRMA fatigue performance remains affected by several methodological limitations. In LAS testing, the lack of a consistent failure criterion can lead to substantially different fatigue-life estimates and limit direct comparisons among materials and studies. Criteria based on a fixed reduction in material integrity may identify failure while highly elastic rubber-modified binders still retain load-carrying capacity, whereas energy-based criteria applied to the same data can rank the same binders differently [1,111]. A consistent failure criterion, potentially based on energy dissipation, is therefore needed to better represent the damage response of highly modified binders and improve comparisons across studies.

Conventional laboratory aging may not fully represent the long-term changes occurring in CRMA during field service, where oxidation of the asphalt phase occurs together with degradation of the rubber network [15,56,57]. This limitation is reflected in field-core comparisons, where nine days of conditioning at 135 °C were required to reproduce the increase in stiffness and reduction in fatigue resistance observed in in-service pavement cores [96]. As set out in Section 5.2, the improved protocols developed in response to this limitation have been validated against bulk rheological equivalence with a reference thermal procedure, which does not demonstrate that the rubber phase has reached a comparable state. Aging protocols for CRMA should accordingly be validated against phase-resolved indicators and against field-aged material, rather than against a laboratory reference alone.

A further limitation is the translation of binder-scale fatigue performance to mixture and pavement behavior. Improvements of up to 116 times the control at 2.5% strain and 45 times at 5.0% strain have been reported at the binder scale [24]. The corresponding values are approximately 1.4 to 10 times at the mixture scale [28,46,100] and approximately 1.4 to 15 times in mechanistic predictions at the pavement scale [41,105,106]. Aggregate structure, air voids, binder-film thickness, and rubber distribution are not represented in binder testing [21,86]. The binder-scale evidence is not itself one-directional, since fatigue lives below that of the base bitumen have also been reported for aged CRMA binders [63], so the difference between scales should be read as a difference in the spread of reported values rather than as a fixed attenuation factor. The upper bound of the mixture-scale range remains provisional for the reason given above. Binder-level parameters, including |G*|sinδ and LAS-derived fatigue life, have also not been systematically validated against observed pavement cracking, and conventional laboratory indicators have shown limited ability to rank field performance [94]. Future research should establish validated relationships between binder-level VECD parameters, mixture fatigue response, and field cracking, while accounting for the strain-dependent fatigue response of CRMA binders. A further gap concerns healing. Rest-period recovery has been shown to extend the fatigue life of CRMA binders through rubber re-swelling and partial restoration of the damaged binder structure [26,27], yet neither the standard LAS protocol nor the mechanistic models reviewed here represents this contribution. Because in-service loading is intermittent, whereas laboratory fatigue testing is continuous, the omission of healing may cause laboratory protocols to understate the field fatigue performance of CRMA, acting in the opposite direction to the scale effects described above. Quantifying the healing contribution under representative rest periods is therefore needed before laboratory fatigue lives can be related to observed pavement cracking. Addressing these limitations will require standardized fatigue-failure criteria, aging procedures representing both degradation processes, protocols that account for healing, and multi-scale validation against observed pavement cracking. Translating the findings into practice will also require work at the specification level, and three steps follow directly from the evidence reviewed here. First, a binder fatigue requirement for highly modified binders should state the failure criterion and the strain amplitude at which it applies, because a single LAS fatigue-life threshold carries no meaning without both. Second, a dosage requirement should state the basis on which rubber content is expressed, since dosages given as a percentage of binder mass and as a percentage of aggregate mass appear in the same literature without distinction and are not interchangeable. Third, where CRMA is specified as a substitute for a polymer-modified binder, acceptance should be supported by mixture-scale fatigue testing rather than by binder-scale screening alone, because the advantage measured on the binder is not reliably reproduced at larger scales. Provisions of this kind would allow multi-scale evidence to be used in material selection and acceptance rather than remaining a laboratory comparison.

A further gap concerns the scale at which aging damage is observed. The aging evidence reviewed here is macroscopic, resting on rheological measurements and oxidation indices obtained by infrared spectroscopy, and it therefore describes the consequences of oxidation rather than the reactions at the rubber–asphalt interface that produce them. Two developments outside the scope of the present search indicate how this gap may be approached. Reactive neural network potentials trained by active learning now allow large-scale molecular dynamics simulation of asphalt oxidation at near-quantum accuracy, and have been used to resolve a sequential dehydrogenation–oxidation-crosslinking reaction network and its temperature dependence in unmodified asphalt [117]. In parallel, in situ nanomechanical visualization has been used to observe nanocracks in aged asphalt during stress relaxation [118]. Neither method has yet been applied to CRMA, where the questions differ: whether oxidative crosslinking of the asphalt phase and degradation of the rubber network alter the interface in ways that change crack initiation, and whether dispersed rubber particles arrest or deflect cracks at the nanoscale. The latter mechanism is assumed in the interpretation of the particle-size evidence reviewed above but has not been observed directly.

These approaches also bear on a limitation of the mechanistic models reviewed above. VECD and fracture-mechanics formulations represent damage through state variables calibrated to binder or mixture tests, and contain no parameter describing the rubber–asphalt interface. If interface damage parameters derived from molecular simulation and nanoscale observation could be related to the damage characteristic curve, a continuous path could be established from interfacial chemistry through nanoscale crack initiation to binder and mixture fatigue. No such framework yet exists, and its development would require experimental validation at each transfer between scales; it is identified here as a research direction rather than as an available method.

A further direction concerns whether aged CRMA can be restored rather than replaced. Rejuvenation of aged rubberized materials has been demonstrated at both scales. Soybean oil applied at 10% by binder weight to plant-produced rubber-modified mixtures conditioned to simulate extended field aging increased disk-shaped compact tension fracture energy by 137% for the five-day aged mixture and by 211% for the ten-day aged mixture, and improved cracking tolerance indices by 22% to 104%, restoring or surpassing the cracking performance of the unaged mixture, while reducing rutting tolerance indices by 52% to 70% [119]. Tire pyrolytic oil brought aged unmodified, polymer-modified and crumb rubber-modified binders to rheological and oxidation-index states close to their unaged condition, with an optimal dosage of 9% [120]. At the mixture scale, reclaimed asphalt rubber pavement has been recycled at 30% into new hot-mix asphalt, with a rejuvenating agent restoring mechanical behavior close to that of a virgin-material reference [121]. Restoration is nevertheless bounded: each binder has a critical aging point beyond which recovery becomes minimal, and rubberized binder retained rejuvenation effectiveness over a longer aging duration than base binder, with the limit occurring later for fatigue life than for high-temperature performance [122].

What this evidence does not establish is the mechanism at the interface, and the gap is narrower and more specific than an absence of interfacial work. The action of bio-based oils on the rubber–asphalt interface has in fact been characterized: such oils alter rubber surface chemistry through sulfur–carbon bond cleavage, radical formation, and grafting, raising surface energy and adsorption and thereby strengthening interfacial interaction, whereas petroleum-derived oils act predominantly as swelling agents with weaker interfacial modification [123]. The two routes have been separated experimentally as chemisorption of bio-derived molecules grafted onto the rubber surface and physisorption of oil absorbed into the particle [124]. Degradation of the rubber network itself can be quantified and related to cracking resistance [72]. Methods for measuring adhesion in these systems are likewise established, including pull-off testing of binders combining crumb rubber with reclaimed asphalt [125] and the digital-image-correlation interface damage factor described in Section 5.2 [71].

What has not been done is to apply this characterization to aged CRMA. In every study above the rubber is unaged: the oils act on virgin crumb rubber to improve compatibility before it enters the binder, not on a rubber–asphalt interface that has already undergone oxidation and network degradation in service. Whether a rejuvenator reaches that aged interface, whether it restores adhesion there or acts only by softening the surrounding matrix, and whether restored adhesion is reflected in recovered fatigue resistance are therefore open questions, even though the materials, the mechanisms and the measurements needed to answer them are all available individually. Combining them would also generate the damage and recovery measures required by the interface parameter identified above, which current damage models do not contain.

## 10. Conclusions

This review critically evaluated the fatigue cracking resistance of CRMA across binder, mixture, modeling, and field scales. The main conclusions are as follows:

1. Crumb rubber improves fatigue resistance through rubber–asphalt interaction and the elastic contribution of undissolved rubber particles. Adequate swelling, dispersion, and bonding can enhance energy dissipation and slow crack propagation, whereas insufficient interaction or aging-related weakening of the rubber–asphalt interface may promote localized damage.
2. No universal optimum exists for crumb rubber content or particle size. Their effects depend on the damage stage evaluated, material scale, dosage basis, and processing method. Binder-scale optimum dosages may exceed mixture-scale values, while the preferred particle size depends on the balance between particle reinforcement and rubber–asphalt interaction.
3. The fatigue benefit of crumb rubber modification is strain-dependent. Improvements are generally greater at 2.5% strain than at 5.0%, and rankings obtained at one strain level may not remain consistent at another. Therefore, fatigue life should be evaluated at strain levels representative of the intended pavement structure.
4. Conventional linear viscoelastic parameters, including |G*|sinδ and the Glover–Rowe parameter, have limited ability to characterize progressive fatigue damage in highly modified CRMA binders because they are obtained under small-strain linear viscoelastic conditions and do not directly represent damage evolution or post-peak integrity. LAS testing combined with VECD analysis provides a more informative binder-level assessment, although fatigue-life estimates remain sensitive to the failure criterion and analysis procedure.
5. Aging generally reduces CRMA fatigue resistance through changes in both the asphalt phase and rubber network. Conventional laboratory aging may not fully reproduce these combined changes during long-term field service, emphasizing the need for aging procedures with greater field relevance.
6. The largest reported fatigue benefits occur at the binder scale and the smallest at the field scale, although the trend is not monotonic and exceptions occur in both directions. Binder-level studies have reported improvements of up to 116 times the control at 2.5% strain and 45 times at 5.0% strain. Mixture-level improvements are generally about 1.4 to 10 times, with the upper bound of that range still provisional. Field performance is commonly comparable to or moderately better than that of modified controls. This difference reflects additional structural, environmental, and construction-related influences at larger scales, together with the use of stronger comparators in field studies. It is not a fixed attenuation factor: fatigue lives below that of the base bitumen have been reported for aged CRMA binders, and a rubber-modified mixture has been ranked above a polymer-modified mixture in beam fatigue testing.

Taken together, these findings support a scale-aware approach to the specification of CRMA. Where the expected in-service strain is low, binder-scale screening at 2.5% strain provides a reasonable basis for material selection, and systems with well-developed rubber–asphalt interaction are the more promising candidates. Where the expected strain is high, the binder-scale advantage should not be assumed to carry over, and selection should be supported by mixture-scale testing. These are statements about test interpretation rather than about pavement thickness. The shear strain imposed on a binder specimen in the dynamic shear rheometer is not equivalent to the strain state of a pavement layer, which also depends on temperature, layer thickness, mixture modulus, interlayer condition, and traffic loading. The amplitudes of 2.5% and 5.0% are accordingly used here as commonly adopted lower- and higher-strain reference conditions and not as proxies for specific pavement structures. Rubber dosages established at the binder scale should not be transferred directly to mixture design, and rubber used as an aggregate substitute should not be compared on a dosage basis with rubber used as a binder modifier. Where CRMA is intended to replace a polymer-modified binder rather than an unmodified one, the comparison should be made against that same modified control, because the reported magnitude of the benefit depends strongly on the comparator selected.

Overall, CRMA can be a viable alternative to polymer-modified asphalt under specific material, processing, and structural conditions. Equivalence, rather than consistent superiority, is the more defensible reading of the present evidence, and it should be read together with the comparator and evidence tier of each underlying study. This is a general observation drawn from the available studies and not a statistically demonstrated equivalence. The field evidence base is small, and the reviewed sections differ in climate, traffic level, pavement structure, crumb rubber processing method, and reference material. The observation therefore applies to the conditions actually investigated, namely wet-process and terminal-blend CRMA surface courses compared against SBS-modified or well-designed conventional sections, and it should not be extended to combinations of processing method, dosage and structure that have not been tested. This conclusion does not weaken the case for crumb rubber modification. Where fatigue performance is equivalent to that of polymer-modified control, the material still diverts end-of-life tires from disposal, so performance parity can provide a reasonable basis for adoption rather than representing a negative outcome, provided that cost, constructability, and material availability are also satisfied. The practical question is therefore not whether CRMA outperforms polymer modification under all conditions, but under which conditions equivalence can be relied upon. Future research should prioritize standardized fatigue-failure criteria, representative aging and strain conditions, and validated multi-scale relationships linking binder-level damage characteristics with mixture fatigue behavior and observed pavement cracking.

## Figures and Tables

**Table 1:** Published healing measurements for rubberized binders and mixtures, classified by scale, protocol, rest variable and reported quantity.

Study	Scale	Protocol	Rest Variable	Reported Quantity
Xu et al. [47]	Binder	Fatigue–healing–fatigue (DSR); base, SBS- and crumb rubber-modified binders at three aging levels	Rest between fatigue blocks	Healing index; regression model from rheological parameters
Subhy and Lo Presti [48]	Binder	Time sweep (DSR) with short rest periods inserted after a set number of load pulses	Rest period (10 s)	Healing potential index from the plateau value of the ratio of dissipated energy change
Xu et al. [49]	Binder	Fatigue–rest–fatigue on pretreated crumb rubber binders, with molecular dynamics simulation	Rest period; healing temperature	Self-healing rate, raised by more than 50% through preswelling before microwave irradiation
Sun et al. [50]	Binder	Fatigue–healing–fatigue over a wide temperature range; aged and bio-oil regenerated terminal-blend rubberized asphalt	Temperature	Regeneration recovery percentage
Ayyar et al. [51]	Mixture	Four-point bending at 20 and 0 degrees C under continuous and rest-period loading; crumb rubber-modified, elastomer-modified and unmodified mixtures	Rest between load cycles	Fatigue life by stiffness and by dissipated- energy criteria; ranking of mixtures
Souliman et al. [52]	Mixture	Four-point bending with rest periods following AASHTO T321; asphalt rubber mixture	Applied strain and rest period	Stiffness-ratio model; endurance limit
Huang and Huang [53]	Mixture	Four-point bending of nine modified and two base mixtures at equal binder content	Healing considered in mixture design	Qualitative grading of healing performance
Noojilla and Kusam [54]	Mixture	Microwave-induced healing of notched specimens; dense- and gap-graded, including gap-graded rubberized	External microwave heating, not ambient rest	Recovery of peak load, fracture energy and crack area

**Table 2:** Comparative fatigue and cracking performance of crumb rubber asphalt produced through wet, dry, and intermediate incorporation processes.

Study	Mixture and CR Dosage	Fatigue Cracking Evaluation Method	Wet-Process Outcome	Dry-Process Outcome	Comparative Conclusion
Xie and Shen [46,82]	Stone-matrix asphalt; 10% CR by binder mass, 30 mesh (+4.5% trans-polyoctenamer, dry route)	Direct-tension S-VECD (mixture) + improved LAS (binder)	Fatigue life greater than the unmodified binder but lower than the terminal-blend and SBS-modified mixtures.	Similar to, or slightly above, the wet-process mixture	Dry ≈ wet, both above virgin binder, below terminal blend and SBS; binder LAS ranking mirrored mixture ranking
Chavez et al. [83]	Gap-graded; 8.6% high-viscosity CR binder (18% rubber) by aggregate mass	Indirect Tensile Fatigue Test (ITFT) + Semi-Circular Bend (SCB) Test	Satisfactory fatigue behavior (reference route)	Dry inferior to semi-wet at equal digestion time	Semi-wet matched wet after 30 min; dry remained inferior
Feiteira Dias et al. [80]	Gap-graded; fine granulate rubber, ≈20% by binder mass	Four-point bending (EN 12697-24) [92]	Improved fatigue vs. conventional mixture (benchmark)	Same improved fatigue tendency as the wet process	Dry fatigue at wet-process level; >190 °C plant temperature impairs rubber contribution
Picado-Santos et al. [84]	Gap-graded; 20% CR by binder mass	Four-point bending test + 8-year field survey	Wet-process benchmark from companion studies	Robust field behavior after 8 years and approximately 1.2 million equivalent single-axle loads (ESALs)	Dry fatigue tendency equal to wet; >190 °C plant temperature impairs fatigue
Riekstins et al. [86]	Dense-graded; wet 25% CR, dry 41% CR by binder mass (aggregate substitute)	Four-point bending test	Best fatigue of all mixtures tested, above the PMB reference	Cohesion-deficient; exceeded 50/70 only through its 6.875% binder content	Wet superior where dry uses high-dosage aggregate substitution
Li et al. [89]	30% RAP mixtures; activated rubber pellets (semi-wet)	Indirect tensile asphalt cracking test (IDEAL-CT)	Restored RAP cracking resistance (benchmark)	Below wet; semi-wet ARP equivalent to wet with better workability	ARP restored RAP cracking resistance to fresh asphalt–rubber level

**Table 3:** Field fatigue performance of crumb rubber-modified asphalt pavements.

Reference	Location	CR Type & Process	Traffic/Exposure	Evidence	Comparator	Observed Fatigue Outcome
Powell and Rodezno [93]	NCAT Test Track, Alabama, USA	11% ground tire rubber, dense-graded (wet); gap-graded asphalt rubber	20 M ESALs over two research cycles	Instrumented full-scale sections	SBS-modified; 20% RAP control	Dense-graded CRM: remained crack-free and performed comparably to SBS; gap-graded asphalt rubber: 0% cracked lane area after 16 million ESALs
Pant et al. [94]	5 projects, Louisiana, USA	Wet (16-mesh) and dry (PlusRide), R-80	In service > 8 years	Observed distress (pavement management system surveys)	Conventional and SBS-modified controls	Statistically comparable or better alligator/transverse cracking and pavement condition than controls; SBS outperformed CRM in some sections
Farina et al. [90]	Local roads, Michigan, USA	Pre-swollen crumb rubber (dry-added)	5 years	Visual survey and Disk-shaped Compact Tension (DCT) testing	Adjacent control lanes	Control-lane transverse cracks terminated at boundaries of pre-swollen crumb rubber lanes, indicating retarded crack propagation
Sierra-Carrillo de Albornoz et al. [95]	3 highways, Spain	CRMA binder, asphalt concrete for very thin layers surface course (wet)	63 months, heavy traffic	Field-recovered cores (fatigue, stiffness)	SBS-modified surface courses	Fatigue/stiffness evolution comparable to SBS; both increasingly fatigue-susceptible after 20–40 months strain-hardening period
Cherkaoui et al. [96]	Highway, Spain	Gap-graded CR mixture	60 months	Cores vs. laboratory aging	Short-term aging protocols	9 days at 135 °C required to replicate field stiffening and fatigue deterioration, evidencing a strong aging-fatigue interaction

**Table 4:** Mechanistic modeling of fatigue cracking in CRMA.

Reference	Scale	Distress/Loading	Modeling Approach	Data Used for Calibration	Fatigue-Related Finding
Gao et al. [98]	Binder	Fatigue (crack growth)	Fatigue crack-growth model combining a pseudo-J-integral Paris law with a damage-mechanics-based DSR-C crack-length formulation.	DSR time-sweep tests of aged and unaged unmodified and polymer-modified binders.	Paris-law coefficients were largely independent of loading frequency and strain amplitude, supporting their use as material-specific parameters; Fatigue crack growth accelerated with aging and decreasing temperature.
Shi et al. [17]	Binder	Fatigue (crack growth)	Crack growth model: fatigue crack density evolution with a modified Paris’ law.	DSR fatigue tests on SBS/CR binders (30–40% CR).	1% SBS + 40% CR gave the best crack-growth resistance; crack-density evolution reliably captures damage.
Al-Khateeb et al. [99]	Binder	Fatigue (binder life)	Continuum-damage model: LAS-based VECD fatigue-life prediction.	LAS on PAV-aged CR warm-mix binders (0–20% CR; Sasobit/Zycotherm).	Predicted fatigue life (Nf) rises consistently with CR content up to 20%.
Imoh et al. [100]	Mixture	Fatigue + rutting	Combined mechanistic–empirical model: viscoelastic + viscoplastic + S-VECD fatigue damage.	IDT creep and relaxation tests, Marshall testing, and cyclic IDT fatigue tests (0–25% CR).	Reproduces the experimental fatigue optimum at 10–15% CR and accelerated degradation at ≥20% CR.
Zhi et al. [106]	Pavement	Fatigue (crack initiation)	Finite element damage mechanics fatigue simulation.	CR-modified asphalt-concrete wearing course fatigue testing; pavement analysis based on 3000 load cycles/day.	Predicted crack-initiation life of 7.6 years for the CR-modified wearing course vs. 5.4 years for the unmodified control mixture.
Kocak & Kutay [41]	Pavement	Fatigue (pavement life)	PP-VECD-based continuum-damage modeling integrated with AASHTOWare Pavement ME.	Push–pull fatigue and dynamic modulus (wet, terminal-blend, devulcanized rubber).	Predicted fatigue life increased by three to fifteen times, while maintenance initiation was delayed from 10.5 to 154.8 months.
Castillo et al. [105]	Pavement	Fatigue (pavement life)	Structural response analysis: 3D-Move viscoelastic simulation.	Laboratory characterization of terminal-blend binders containing 10% and 50% tire rubber.	Predicted pavement fatigue life was five to seven times greater than that of an equivalent structure containing unmodified asphalt.
Thives et al. [103]	Pavement	Fatigue (thickness design)	Empirical fatigue law used in structural thickness design.	Four-point bending stiffness and fatigue tests.	Asphalt rubber surface layer can be ≥50% thinner than a conventional mixture for the same design life.
Thives et al. [104]	Pavement	Reflective cracking (fatigue)	ANSYS 10.0 Multiphysics-based finite element analysis using von Mises strain-based fatigue relationships to predict reflective cracking life.	Four-point bending + reflective cracking device.	The predicted reflective cracking life of asphalt–rubber overlays was nearly eight times that of conventional overlays.
Jin et al. [107]	Pavement	Fatigue cracking, rutting, and International Roughness Index (IRI)	AASHTOWare Pavement ME 20-year distress prediction.	Dynamic modulus, IDT fracture energy, DSR (dry process).	Over a 20-year design life, the CRM pavement showed less bottom-up fatigue cracking, rutting, and lower IRI than conventional HMA.

**Table 5:** Interpretation of contrasting fatigue responses associated with crumb rubber dosage, particle size, and strain amplitude.

Studies	Evidence Basis (Scale/Test)	Contrasting Findings	Reason for the Difference	Interpretation
Rubber dosage				
Kök et al. [28]; Shi et al. [17]	Binder/crack-propagation testing vs. binder/DSR fatigue with modified Paris law	Crack-propagation resistance decreased above 4% CR in one study, whereas fatigue resistance improved with increasing CR content up to 40% in an SBS/CR composite binder	Kök et al. measured crack propagation only, whereas Shi et al. used an index integrating initiation and propagation in a composite binder	A dosage that delays crack initiation does not necessarily provide the same resistance to crack propagation. The two findings represent different stages of fatigue damage
Shi et al. [17]; Imoh et al. [100]	Binder/DSR fatigue vs. mixture/cyclic IDT with S-VECD	Binder-level fatigue resistance improved at high CR contents, whereas mixture- level performance was highest at 10–15% CR and declined at 20% or more	Mixture tests include aggregate structure, compaction, and cohesion, which are absent from binder tests	Because these mixture-level constraints are not represented in binder testing, the binder-level optimum may exceed the mixture-level optimum. Binder-level dosages should not be transferred directly to mixture design
Riekstins et al. [86]	Mixture/four-point bending	A wet-process mixture containing 25% CR showed higher fatigue resistance than a dry-process mixture containing 41% CR, which exhibited lower cohesion	Rubber modified the binder in the wet process but served partly as an aggregate substitute in the dry process	Rubber dosages should not be compared directly when the rubber performs different functions within the mixture
Particle size				
Singh et al. [11]; Wen et al. [12]	Binder/LAS (both studies)	Coarser particles (#30–40) outperformed finer particles (#60–80) in a wet-process binder, whereas in terminal- blend binders fatigue-life improvement increased from 50% (30–50 mesh) to 70% (50–100 mesh) and 80% (100–200 mesh)	Wet processing preserved more discrete rubber particles, whereas terminal-blending promoted greater rubber dissolution and interaction	The preferred particle size depends on the processing method and the extent of rubber–asphalt interaction
Singh et al. [11]; H. Wang et al. [21]	Binder/LAS vs. mixture/fatigue and crack-growth testing	Coarser particles produced longer fatigue life at binder level, whereas 80-mesh rubber improved mixture fatigue life and reduced crack-growth rates	Rubber in a mixture is confined within a thin binder film where the uniformity of particle distribution influences the local stress field	In mixtures, more uniform distribution of finer particles may reduce localized stress concentrations, outweighing the contribution of larger individual particles
Strain amplitude				
Wen et al. [12]; S. Chen et al. [24]	Binder/LAS (both studies)	The terminal-blend advantage of 50–80% at 2.5% strain largely disappeared at 5.0% strain, whereas a 12% terminal-blend binder retained a fatigue-life ratio of 18 at 5.0% strain	The low-strain benefits differed substantially between the two studies, resulting in different levels of remaining benefit at 5.0% strain	Fatigue life should be reported at both strain amplitudes because rankings obtained at one strain level may not apply at the other

**Table 6:** Reported magnitude of CRMA fatigue benefits across evaluation scales, with the evidence tier and comparator applying at each scale.

Scale	Tier	Method	Comparator	Reported Gain	Important Consideration
Binder	Tier 1	LAS with VECD; time sweep	Unmodified base binder, same PG grade	From below the control to 116× at 2.5% strain; 45× at 5.0%	Widest range, and it runs in both directions: aged CRMA binders have also been reported with fatigue lives below that of the base bitumen. Depends strongly on strain level, failure criterion, and aging state. Aggregate structure is absent, so the value bounds material potential rather than pavement performance
Mixture	Tier 1, with one Tier 2 entry	Direct-tension S-VECD; four-point bending; cyclic IDT	Unmodified mixture, similar gradation	About 1.4 to 10×	Upper bound still requires confirmation against a repeated-load fatigue result. Gradation, air voids, film thickness and binder content also matter. Studies that report the comparison as a ranking against a named control, rather than as a ratio, are not included in this range
Model	Tier 1 inputs, predicted output	PP-VECD with Pavement ME; 3D-Move; FE damage mechanics	Unmodified or softer-grade structure, similar geometry	About 1.4 to 15×	Prediction, not measurement. Calibration and transfer functions may not capture rubber morphology, healing, or the aging-fatigue interaction
Field	Tier 1 on cores; observed distress	Instrumented sections; distress surveys; core testing	SBS-modified or well-designed conventional sections	Comparable to moderately better	The comparator is stronger here than at other scales, so the smaller apparent benefit is not due to scale alone. Traffic, climate and construction quality also contribute

**Table 7:** Studies in which more than one failure criterion, or more than one fatigue approach, was applied to the same data set. Studies applying a single criterion are recorded so that comparisons conditional on it can be identified: [27] uses the peak in stored pseudo-strain energy, [35] the 35% reduction criterion as standardized for LAS, and [44] C = 0.5 at the mixture scale. No study listed reports a numerical ratio between fatigue lives obtained under different criteria for the same material.

Study	Materials	Criteria or Approaches Compared on the Same Data	Ranking Preserved?
[113]	Unmodified, polymer-modified and asphalt rubber binders and mixtures	Five LAS failure criteria, plus |G*|sinδ; several aging levels and temperatures	No; LAS-PSE does not show the AR improvement, maximum shear stress and integrity-curve criteria do
[111]	Hybrid binder with recycled LDPE and crumb rubber	35% reduction in |G*|sinδ against peak stored pseudo- strain energy and dissipated energy	No; the 35% criterion under-rates the hybrid binder
[1]	Terminal-blend crumb rubber and SBS composite modified binder	Four time-sweep criteria (50% modulus decay, phase-angle peak, S × N peak, 20% dissipated-energy-ratio deviation) with LAS-VECD and a fracture model	Partly; the phase-angle-peak criterion is unsuitable for this material
[114]	Crumb rubber, waste cooking oil and SBS modified binders	G^R against W_sum^R within VECD; LAS and time sweep; several temperatures	Yes for the sign, no for transfer; only W_sum^R unifies methods and temperatures
[110]	Warm asphalt rubber, 60/70 penetration binder with 18% 40-mesh crumb rubber	|G*|sinδ, LAS, indirect tensile fatigue, four-point bending, mortar shear fatigue	Partly; LAS, mortar shear and four-point bending agree, the other two do not
[115]	Seven modified binders including crumb rubber and terminal blend	Four failure criteria across time sweep, LAS and elastic recovery	Yes; elastomeric binders rank highest throughout
[2]	Long-term aged crumb rubber-modified bitumen with warm-mix additives	Superpave fatigue parameter and Glover–Rowe against time-sweep dissipated energy and LAS-S-VECD	No; the first two do not characterize the modified binders adequately
[116]	Crumb rubber-modified binders	Time-sweep failure criteria under both stress- and strain-controlled loading	Condition dependent; the stress- or strain-to-failure relationship differs by control mode

## Data Availability

No new data were created or analyzed in this study. All data discussed are available in the cited publications.

## Abbreviations

AASHTO, American Association of State Highway and Transportation Officials; APAO, amorphous poly-alpha-olefin; ARP, activated rubber pellets; ASCE, American Society of Civil Engineers; CR, crumb rubber; CRM, crumb rubber modifier; CRMA, crumb rubber-modified asphalt; CRMB, crumb rubber-modified bitumen; CRNf, change rate of fatigue life; DCT, disk-shaped compact tension; DSR, dynamic shear rheometer; DVR, devulcanized rubber; FE, finite element; FTIR, Fourier-transform infrared spectroscopy; IDEAL-CT, indirect tensile asphalt cracking test; IDT, indirect tensile; IRI, International Roughness Index; ITFT, indirect tensile fatigue test; LAS, linear amplitude sweep; LVE, linear viscoelastic; ME, mechanistic–empirical; MSCR, multiple stress creep and recovery; NCAT, National Center for Asphalt Technology; PAV, pressure aging vessel; PG, performance grade; PMB, polymer-modified bitumen; PP, push–pull; PRISMA, Preferred Reporting Items for Systematic Reviews and Meta-Analyses; RAP, reclaimed asphalt pavement; RAR, reacted and activated rubber; RTFOT, rolling thin-film oven test; SBS, styrene–butadiene–styrene; SCB, semi-circular bend; SHRP, Strategic Highway Research Program; S-VECD, simplified viscoelastic continuum damage; TB, terminal blend; UV, ultraviolet; VECD, viscoelastic continuum damage.

## Appendix A

This appendix reports the search strategy in the form requested during review: the strings themselves, the database against which each was executed, the date of execution, and the number of records returned. It also reports a recall test of both the published and the revised strategy, and the limitations that remain.

### Appendix A.1. Strategy Used in the Original Submission

The following Boolean strategy was used for the searches reported in Section 2, conducted in May 2026:

((“crumb rubber” OR “crumb rubber modified” OR “CRMB” OR “rubberized asphalt” OR

  “rubber modified asphalt” OR “crumb rubber modified bitumen” OR
  “crumb rubber modified asphalt”)
 AND
 (“fatigue” OR “fatigue cracking” OR “fatigue resistance” OR “fatigue life” OR
  “fatigue performance” OR “fatigue damage” OR “cracking resistance”)
 AND
 (“asphalt binder” OR “bitumen” OR “modified binder” OR “modified bitumen”)
 AND
 (“DSR” OR “dynamic shear rheometer” OR “LAS” OR “linear amplitude sweep” OR “VECD” OR
  “S-VECD” OR “viscoelastic continuum damage” OR “aging” OR “long-term aging” OR
  “aged bitumen” OR “particle size” OR “wet process” OR “dry process” OR
  “terminal blend” OR “elastic recovery” OR “damage model” OR “mechanistic model” OR
  “field performance”))

It is retained here because the results reported in Section 2 were obtained with it. Two properties of this strategy limit its reach across evaluation scales. The third block makes a binder term mandatory, so that a record had to contain binder-level vocabulary to be retrieved at all; and the fourth block, which supplies the methodological and topical vocabulary, contains no mixture-scale test name, with “beam fatigue”, “four-point bending”, “indirect tensile fatigue”, “semi-circular bend”, “overlay test”, “asphalt mixture”, “asphalt concrete” and “field cracking” all absent. The two act in the same direction. The consequence is quantified in Appendix A.4.

### Appendix A.2. Revised Scale-Specific Strategy

Three strings were constructed, one for each evaluation scale compared in this review, and are used as a union rather than as a single filter. Two blocks are common to all three:

   [CR]  (“crumb rubber” OR “ground tire rubber” OR “GTR” OR “tire rubber” OR
      “scrap tire” OR “waste tire” OR “rubberized asphalt” OR “rubberised asphalt” OR
      “asphalt rubber” OR “rubber modified asphalt” OR “rubber modified bitumen”
OR
      “crumb rubber modified” OR “CRMB” OR “devulcanized rubber” OR
      “reacted and activated rubber” OR “terminal blend”)
[FA]  (“fatigue” OR “fatigue cracking” OR “fatigue life” OR “fatigue resistance” OR
      “fatigue performance” OR “fatigue damage” OR “cracking resistance” OR
      “crack propagation” OR “crack growth” OR “cracking performance” OR
      “damage characteristic” OR “reflective cracking” OR “bottom-up cracking” OR
      “top-down cracking”)

**S1, binder scale.**

[CR] AND [FA] AND

(“asphalt binder” OR “bitumen” OR “binder” OR “mastic” OR “DSR” OR
 “dynamic shear rheometer” OR “LAS” OR “linear amplitude sweep” OR “time sweep” OR
 “VECD” OR “S-VECD” OR “viscoelastic continuum damage” OR “Glover-Rowe” OR
 “rheological” OR “rheology” OR “complex modulus” OR “phase angle”)

**S2, mixture scale.**

[CR] AND [FA] AND

(“asphalt mixture” OR “asphalt concrete” OR “asphalt mix” OR “hot mix asphalt” OR
 “HMA” OR “warm mix asphalt” OR “gap-graded” OR “open-graded” OR “dense-graded” OR
 “stone matrix asphalt” OR “SMA” OR “beam fatigue” OR “four-point bending” OR
 “flexural fatigue” OR “indirect tensile fatigue” OR “IDT” OR “semi-circular bend” OR
 “SCB” OR “overlay test” OR “dry process” OR “wet process” OR “mixture performance”)

**S3, field and pavement scale, including mechanistic modeling.**

[CR] AND [FA] AND

(“field performance” OR “field cracking” OR “field evaluation” OR “field section” OR
 “field trial” OR “field core” OR “test track” OR “in-service pavement” OR
 “pavement performance” OR “pavement section” OR “long-term pavement performance” OR
 “LTPP” OR “Pavement ME” OR “mechanistic-empirical” OR “finite element” OR
 “3D-Move” OR “pavement design” OR “pavement thickness” OR “overlay” OR
 “flexible pavement” OR “service life”)

Two design changes distinguish these from the published strategy. Mandatory binder vocabulary was removed and replaced by scale-appropriate vocabulary, so that a mixture or field study is retrievable without using binder terms; and the number of mandatory blocks was reduced from four to three, the four-way conjunction having proved over-restrictive in the test reported in Appendix A.4. Modeling vocabulary was placed in S3 rather than in a fourth string, because the modeling studies reviewed in Section 7.2 predict performance at the pavement scale.

### Appendix A.3. Execution

The three strings were executed with the filters written into the query rather than applied through the interface, so that the query can be reproduced by pasting the string alone. The form used was:

        TS = ([CR]) AND TS = ([FA]) AND TS = ([scale block])
AND PY = (1990–2026) AND DT = (Article OR Review) AND LA = (English)

Records were exported as tab-delimited Full Record files including abstracts and DOIs.

**Table A1:** Scale-specific search strings, database, search date, and number of records returned. Pairwise overlaps were 328 records between S1 and S2, 135 between S1 and S3, and 122 between S2 and S3; 74 records were returned by all three strings.

String	Scale	Database	Search Date	Records Returned
S1	Binder	Web of Science Core Collection	28 September 2026	720
S2	Mixture	Web of Science Core Collection	28 September 2026	467
S3	Field and pavement, including mechanistic modeling	Web of Science Core Collection	28 September 2026	217
Union of S1–S3	All three	Web of Science Core Collection	28 September 2026	893 (simple total 1404; 511 duplicate records removed)

Because S1 returned the same count with and without the document-type filter, the filter was verified rather than assumed: replacing DT = (Article OR Review) with DT = (Review) reduced the return from 720 to 37 records, confirming that the restriction was applied and that 720 is the unrestricted count for that string.

For re-execution in Scopus, the equivalent syntax is:

TITLE-ABS-KEY ( [CR] AND [FA] AND [scale block] )

AND PUBYEAR > 1989
AND ( LIMIT-TO ( LANGUAGE , “English” ) )
AND ( LIMIT-TO ( DOCTYPE , “ar” ) OR LIMIT-TO ( DOCTYPE , “re” ) )

The re-execution reported in Table A1 was carried out in Web of Science Core Collection rather than in Scopus, which was the primary database of the original search. This is a deviation from Section 2 and is stated as such: the counts in Table A1 are Web of Science counts and are not interchangeable with Scopus counts. A preliminary execution of the same three strings against an open bibliographic database, carried out before the Web of Science access described above became available, returned 566, 547 and 184 records for S1, S2 and S3, respectively, and identified the five studies admitted at the first revision.

### Appendix A.4. Recall Test

The reach of a search strategy can be tested directly against the reference list the review already contains: a strategy that cannot retrieve the studies the review cites is not reproducible. Both strategies were tested this way.

**Table A2:** Recall of the revised and published search strategies, measured against the reference list of the submitted manuscript.

Step	Count
References in the submitted manuscript	95
Of these, carrying a DOI	87
Of these, indexed in Web of Science Core Collection	81
Retrieved by the revised strings S1–S3	66
Recall of the revised strategy	81% (66 of 81)
Recall of the published string on a comparable sample	40%
Recall of the published string at the mixture, field and modeling scales	1 of 13

The 21 studies that the revised strings did not retrieve were examined individually by querying their DOIs against the same database. Fifteen are present in Web of Science but were not returned by the strings, and are therefore a limitation of the vocabulary. Six are not indexed in Web of Science Core Collection at all and cannot be retrieved by any string executed there; they are agency reports, standard test methods, and journals outside that index. This division is the reason the supplementary-source path shown in Figure 1 is necessary rather than optional, and it is reported here because the original submission did not quantify it.

### Appendix A.5. Screening of the Retrieved Records

Of the 893 records in the union, 66 were already cited in the submitted manuscript. The remaining 827 were screened against the inclusion and exclusion criteria stated in Section 2. Thirty-eight were removed as outside the subject scope, leaving 789 candidate records. Records were ranked by the number of review-relevant topics present in the title and abstract, and screening proceeded from the highest-ranked groups; 236 records were assessed at title and abstract level, and 29 studies were admitted to the synthesis at this revision. Full texts were obtained for all 29, and each was read against the statements attributed to it; one of those checks led to a correction in Section 9, where a quantitative claim was found not to be supported by the source cited for it and was replaced by the qualitative statement that the evidence does support.

Screening of the remaining candidate records is not complete. The records not yet assessed are those ranked lowest by the same criterion, and they are concentrated in topic groups already represented in the synthesis. This is stated explicitly rather than implied by an unqualified selection count.

### Appendix A.6. Limitations of the Search

Four limitations of the search and screening process are reported for completeness.

First, the re-execution was carried out in a single database. The counts in Table A1 are therefore not a multi-database yield, and the strings have not been executed in Scopus, Google Scholar, ScienceDirect, or the ASCE Library, the sources named in Section 2.

Second, the recall test in Table A2 uses the title, abstract, and keyword fields of the reference list as the basis for matching. It is an approximate test, and it is conservative in one direction and optimistic in another: a record may be retrievable through fields not tested here, while the sample is the review’s own reference list and therefore cannot reveal studies absent from both the search and the reference list.

Third, screening of the candidate pool is incomplete, as stated in Appendix A.5.

Fourth, the position on full texts differs between the studies admitted at this revision and the reference list as a whole. All 29 studies admitted here were read in full. For the reference list as a whole, however, 52 sources were assessed from their abstracts, and 36 could not be assessed at all, which is what limits the grading reported in Appendix B.

## Appendix B

Reviewers asked for a qualitative indication of study quality, since this review applies neither meta-analysis nor a formal risk-of-bias instrument. Table A3 grades the reviewed studies by the completeness of the experimental design and the level of variable control, distinguishing controlled experiments from exploratory ones. Whether the failure criterion is reported is treated separately in Section 8.2 and Table 7, because it can be established only for the studies that state the criterion.

Three limits apply to the grading and are stated so that it is not read as more than it is.

First, the grade depends on the evidence available to us about each study. Full texts were obtained for 37 of the reviewed sources; for 52, the assessment rests on the abstract, which reports results more fully than design; and for 36, neither an abstract nor a full text could be obtained, so no grade was assigned. The last group is listed rather than omitted, because its size is itself part of the answer.

Second, an abstract-based grade is provisional. The absence of a stated control in an abstract does not establish that the study had none.

Third, the grade is independent of whether this review has used the study correctly. That is recorded separately in the citation-verification ledger maintained for this manuscript, and the two do not always agree: several grade A and B studies carry a partial verification, meaning that the present review states more than the source supports at that point. Those are the places where heterogeneity is most likely to bias a conclusion, and quantitative values are attributed only where the grade and the verification agree.

**Table A3:** Qualitative grading of the reviewed studies by completeness of experimental design and level of variable control. The grade describes the study, not its result; a grade C study may be correct and a grade A study may be narrow.

Grade	Definition	*n*	References
A	Controlled experiment: a comparator is present, and one variable is varied while the others are held constant	22	[11,24,49,52,53,65,66,68,72,73,78,97,103,110,113,114,115,120,121,123,124,125]
B	Controlled comparison: a comparator is present, but several variables move together	33	[1,2,18,19,28,39,41,45,46,47,48,50,51,54,55,63,64,67,69,70,71,76,84,86,94,95,100,104,105,112,116,119,122]
C	Exploratory: the comparator is weak or absent, or a single condition or material is observed	26	[10,14,22,26,27,31,40,44,58,59,60,61,81,82,83,87,88,96,98,99,101,102,107,108,111,117]
D	Background and methodology: reviews, standards and procedural sources rather than experiments	8	[3,4,5,6,15,35,75,92]
Not assessable	No abstract or full text could be obtained, so the design cannot be judged	36	[7,8,9,12,13,16,17,20,21,23,25,29,30,32,33,34,36,37,38,42,43,56,57,62,74,77,79,80,85,89,90,91,93,106,109,118]
